# Supplementary material for: Efficacy and safety of anakinra in adults presenting deteriorating respiratory symptoms from COVID-19: A randomized controlled trial
Source: PLoS One. 2022 Aug 4;17(8):e0269065. doi: 10.1371/journal.pone.0269065 (PMC9351999; doi:10.1371/journal.pone.0269065)
Supplement: S1 File — (DOC) [file pone.0269065.s001.doc]

| SPONSOR:  University Hospital Center of Tours  2 Boulevard Tonnellé  37044 Tours cedex 9 |  |
| --- | --- |
| **CLINICAL RESEARCH PROTOCOL** | |
| **STUDY CODE** | PHRCN2020-AAV/ANACONDA-COVID-19 |
| **PROJECT TITLE (English version)** | **Efficacy and safety of ANAkinra during Adult « COroNa virus Disease-19 » with Aggravating respiratory symptoms: a multicenter open-label controlled randomized trial** |
| **PROJECT TITLE (French Version)** | Efficacité et tolérance de l’ANAkinra chez les adultes atteints de « COroNa virus Disease-19 » présentant une Aggravation des symptômes pulmonaires: essai contrôlé randomisé multicentrique, en ouvert |
| **ACRONYM** | **ANACONDA-COVID-19** |
| **COORDINATING INVESTIGATOR** | **AUDEMARD-VERGER Alexandra, MD-PhD** |
| **VERSION NUMBER** | 1.0 |
| **DATE** | 13/04/2020 |
| **EudraCT NUMBER** | 2020-001734-36 |
| **REGISTRATION NUMBER** | DR200111 |
| **FUNDING** | CHRU of Tours / Private funding/SOBI |

**HISTORY OF PROTOCOL AMENDMENTS**

| **Version number (after amendment)** | **Date** | **Amendment justification** |
| --- | --- | --- |
|  |  |  |
|  |  |  |
|  |  |  |
|  |  |  |
|  |  |  |

**MAIN CORRESPONDENTS OF THE STUDY**

| **Coordinating Investigator**  **Dr. Alexandra AUDEMARD-VERGER, MD-PhD**  Department of Internal Medicine and Clinical Immunology  University Hospital Center of Tours  Phone : + 33 2 47 47 98 10  E-Mail : [a.audemardverger@chu-tours.fr](mailto:a.audemardverger@chu-tours.fr) | **Sponsor**  University Hospital Center of Tours  **Mr. Julien LE BONNIEC**  Research Director  2 Bd Tonnellé – 37044 Tours Cedex 9  Phone : [+33 2 18 37 08 01](http://annutel/public/annuaire/V7/Resultat.php?tel=70801)  E-Mail : j.lebonniec@chu-tours.fr |
| --- | --- |
| **Other collaborator(s) (industrial)**  **Swedish Orphan Biovitrum SOBI**  **Dr. Corinne GANDOSSI, MD**  Medical Director France & North Africa of SOBI  Tour Pacific – Aile Ouest – 2^ème^ étage  11-13 Cours Valmy 92800 PUTEAUX Phone : +33 1 85 78 03 40  Fax 01 85 78 03 41  Mobile : 06 12 59 59 44  E-Mail : corinne.gandossi@sobi.com |
| **Methodology, Biostatistics and Data Management Centre :**  Unit manager : **Pr. Bruno GIRAUDEAU**  Phone : +33 2 34 37 96 57  INSERM CIC-P 1415, University Hospital Center of Tours  2, bd Tonnellé - 37044 Tours Cedex 9  E-Mail: bruno.giraudeau@univ-tours.fr  Methodologist**: Dr. Agnès CAILLE**, MD, PhD  Data Manager : **Mrs**. **Frédérique MUSSET** | **Coordinating pharmacy**  Hospital pharmacy, University Hospital Center of Tours  Unit manager: **Mr. Philippe MEUNIER**  Pharmacist**: Dr. Hélène BOURGOIN**  Phone : +33 2 47 47 93 90  E-Mail : h.bourgoin@chu-tours.fr |
| **Coordination and monitoring**  Delegation for Clinical Research and Innovation (DRCI), Promotion & Quality Control  Project manager: **Mr. Mohammed Aït AMER MEZIANE**  Clinical Research Assistant: **Mrs. Elodie MOUSSET**  Phone : +33 2 47 47 46 65  E-Mail : e.mousset@chu-tours.fr | **Clinical research vigilance unit**  Vigilance unit for biomedical research, University Hospital of Tours.  Unit manager: **Dr.** **Annie-Pierre JONVILLE-BERA**  Vigilance expert: **Dr. Céline LENGELLÉ/Dr Marie-Sara AGIER**  Phone : +33 2 47 47 80 37  E-Mail : [uvrb@chu-tours.fr](mailto:uvrb@chu-tours.fr) |
| **Scientific committee**  **Pr. Arsène MEKINIAN**  Internal Medicine Department  Hospital Saint Antoine, APHP, Paris.  E-Mail : [arsene.mekinian@aphp.fr](mailto:arsene.mekinian@aphp.fr)  **Pr. Achille AOUBA**  Internal Medicine Department  University Hospital Center of Caen  E-Mail : aouba-a@chu-caen.fr  **Pr. Hervé WATTIER**  Department of Immunology [University](https://www.univ-tours.fr/sciences-de-la-vie-et-de-la-sante/ea-7501-erl-cnrs-7001-groupe-innovation-et-ciblage-cellulaire-gicc--576173.kjsp?RH=ACCUEIL) Hospital of Tours  E-Mail : [watier@med.univ-tours.fr](mailto:watier@med.univ-tours.fr) | **Data and Safety Monitoring Board**  **Dr. Bénédicte LEBRUN-VIGNE**S  Biological Pharmacology Department  Hôpital de la Pitie Salpetrière, APHP  E-Mail : [benedicte.lebrun-vignes@aphp.fr](mailto:benedicte.lebrun-vignes@psl.aphp.fr)  **Pr. Sophie GEORGIN-LAVIALLE**  Internal Medicine Department  Centre de Références des Maladies AutoInflammatoires et des Amyloses inflammatoires (CEREMAIA)  Hospital Tenon, APHP, Paris  E-Mail : [sophie.georgin-lavialle@aphp.fr](mailto:sophie.georgin-lavialle@aphp.fr)    **Dr. Jean-Benoit HARDOUIN**  Department of Clinical Pharmacy - Public Health - Epidemiology – Biostatistics  CHU de Nantes  E-Mail : [Jean-Benoit.Hardouin@univ-nantes.fr](mailto:Jean-Benoit.Hardouin@univ-nantes.fr)  **Pr. Renaud VERDON**  Department of Infectious Diseases  CHU de Caen  E-Mail : Verdon-r@chu-caen.fr |

**PROJECT TITLE**

**Efficacy and safety of ANAkinra during Adult « COroNa virus Disease-19 » with Aggravating respiratory symptoms: a multicenter open-label controlled randomized trial: ANACONDA-COVID-19**

**Study sponsor**

University Hospital Center of Tours**,** 2 Boulevard Tonnellé**,** 37044 Tours cedex 9

Tours,

Sponsor signature

**Julien Le Bonniec**

**Administrative research director**

Research direction

**Coordinating Investigator**

**Alexandra AUDEMARD-VERGER**

Department of internal medicine and clinical immunology

University Hospital Center of Tours

Tours,

Coordinating Investigator signature

**Dr. Alexandra AUDEMARD-VERGER**

**ABSTRACT (English Version)**

| Title | **Efficacy and safety of ANAkinra during Adult « COroNa virus Disease-19 » with Aggravating respiratory symptoms: a multicenter open-label controlled randomized trial: ANACONDA-COVID-19** |
| --- | --- |
| Version | 1.0 |
| Sponsor | University Hospital Center of Tours |
| Coordinating Investigator | Dr AUDEMARD-VERGER Alexandra, MD-PhD |
| Background / Rationale | Severe acute respiratory syndrome coronavirus 2 (SARS-Cov-2) is an emergent coronavirus, first reported in Wuhan, China 2019. The disease is named Corona Virus Disease-19 (COVID-19). So far, the virus has infected more than 1.3 million people all around the world and caused death of more than 74 000 persons (13^th^ April 2020).  **Up to 20% of hospitalized patients need an admission in intensive care medicine to receive ventilation support and the vast majority experienced acute respiratory distress syndrome (ARDS**). To date, there is no efficient therapeutics to prevent or treat COVID-19 related respiratory failure. **Identifying a drug is a major concern and a public health emergency.**  The pathogenesis of COVID-19 encompasses a “**cytokine storm”** which includes pro-inflammatory interleukins (**IL-1β, IL-6**) and tumor necrosis factor (**TNF-α**). Several studies suggest that patients who develop severe respiratory forms of COVID-19 have a deleterious pulmonary and systemic inflammatory cascade. Predictors of mortality from a recent retrospective, multicentre study of 150 confirmed COVID-19 cases in Wuhan, China, included elevated ferritin and elevated C-Reactive Protein (CRP) (mean 126 mg/L in non survivors vs. 34 mg/L in survivors) **suggesting that mortality might be due to virally driven hyperacute inflammation** [4]. It is well known that circulating CRP is produced by hepatocytes under regulatory control from circulating cytokines, in particular IL-6 and IL1β.  **Therefore, we suggest that IL-1β is a potentially therapeutic target to break inflammatory process and to treat patient at risk for developing severe ARDS related to COVID-19**. As a recombinant anti-human IL-1 receptor treatment, Anakinra can specifically binds IL-1R and inhibit signal transduction. For a decade, Anakinra is mainly used to treat patients with rheumatoid arthritis or auto-inflammatory diseases and its safety profile is favorable in such indications.  Moreover, subgroup analysis of data from a phase III randomized controlled trial of IL-1 blockade (Anakinra) in **sepsis**, showed a significant **survival benefit** in patients with hyperinflammation, without increased adverse events as compared to placebo.  Several multicentre, randomized controlled trials of tocilizumab (IL-6 receptor blockade) or sarilumab (IL-6 neutralisation) are ongoing in many countries for COVID-19 patients.  **We, therefore, aimed to study the efficacy and safety of Anakinra plus optimized standard of care compared to optimized standard of care alone, in patients with COVID-19 with aggravating respiratory symptoms and inflammatory component in a multicenter randomized clinical trial.** |
| Primary objective | The main objective of the ANACONDA trial is to assess the **efficacy of Anakinra + optimized Standard of Care (oSOC) as compared to oSOC alone on the condition of patients with COVID-19 infection and worsening respiratory symptoms**. Success defined as patient alive and free of invasive mechanical ventilation (IMV) and free of Extracorporeal Membrane Oxygenation (ECMO) at Day 14. |
| Secondary objectives | 1. **To assess the efficacy of Anakinra + oSOC as compared to oSOC alone on:**  - Treatment success (same definition than primary outcome) up to Day 28 - Patient’s condition as defined by the OMS 7 point scale up to Day 28 - Patient’s mortality up to Day 28 - Patient’s admission in ICU - Pulmonary function (need for ventilator support, SP02, PaO2/FiO2) up to Day 28 - Inflammation parameters up to Day 28  1. **To evaluate the safety profile of Anakinra** 2. **To identify Biomarkers of efficacy of Anakinra.** |
| Study design | ANACONDA study is a French **multicentre**, **open-label**, randomized, and **controlled** superiority trial comparing the administration of optimized standard of care and Anakinra versus optimized standard of care alone in patients hospitalized in a medical unit with COVID-19. After consent, eligible patients will be randomly allocated with a 1:1 ratio to either Anakinra plus optimized standard of care or optimized standard of care alone. **Randomization** (**Day 0**) will be **stratified** on **baseline CRP concentration** (<150 vs. >=150 mg/L), **baseline requirement of oxygen therapy** to maintain Sp02 over 92% (3-6 liters per min vs. 7-10 liters per min), and **corticosteroid** therapy at baseline (< vs. >=0.5mg/kg/day prednisone) (yes vs no). **Patients will be followed up to day 28**. Baseline measurements will include clinical, radiological and biological measurements. Patients will be followed daily during hospitalization and study visits will be performed at **Day 3, Day 10, Day 14 and Day 28**. Due to feasibility concern in the current emergency circumstances, the trial is open labelled (no placebo arm) but the primary outcome is objective thus preventing any detection bias in the trial results.  We plan to recruit **240 patients**, 120 patients per group. In order to allow an early stopping of the trial in relation with efficacy, an **interim analysis** will be performed after the primary outcome has been obtained for **the first 120 patients**. We will use a Pocock’s approach with a p-value boundary of 0.0294 for both the interim and final analysis. Results of the interim analysis will be reviewed by **a data safety and monitoring board** (DSMB) who will provide a recommendation on whether the trial should be carried on or not. The DSMB will also review the safety data on a regular basis (**every 60 included patients**). |
| Primary outcome | The primary endpoint is treatment success at **Day 14**, defined as a **patient alive** and **not requiring any of the following: Invasive mechanical ventilation (IMV) or Extracorporeal membrane oxygenation (ECMO).** |
| Secondary outcomes | **1. Efficacy outcomes :**   - Treatment success (same definition as the Primary outcome) at Day 3, Day 10 and Day 28 - OMS progression scale (on a 7-point ordinal scale): at Day 3, Day 10, Day 14 and Day 28   1. Not hospitalized, no limitations on activities  2. Not hospitalized, limitation on activities;  3. Hospitalized, not requiring supplemental oxygen;  4. Hospitalized, requiring supplemental oxygen;  5. Hospitalized, on non-invasive ventilation or high flow oxygen devices;  6. Hospitalized, on invasive mechanical ventilation or ECMO;  7. Death.   - Overall survival at Day 3, Day 10, Day 14 and Day 28 - Time to ICU admission - Time to ventilatory support (ECMO, invasive mechanical ventilation, non-invasive ventilation, high flow oxygen therapy) - Change in NEWs score from baseline to Day 3, Day 10, Day 14 and Day 28 - Change in HScore from baseline to Day 3, Day 10, Day 14 and Day 28 - Change in inflammatory parameters (CRP, ferritin, D-dimer, fibrinogen, lymphocytes count, platelet count) from baseline to Day 3, Day 10, Day 14 and Day 28 - Hospital length of stay: Time from inclusion to hospital discharge   For those admitted in ICU:   - Need for Vasopressors (yes or no) - If IMV, Evolution of SpO2/FIO2 - If IMV, Evolution of PaO2/FiO2 ratio - ICU length of stay: Time from admission in ICU to ICU discharge   Secondary outcomes of the trial will be updated according to the COS for COVID-19.  **2. The safety outcomes :**  Occurrence of serious adverse events during the study, including bacterial infection, septic shock, hepatitis (SGOT/SGPT, alkaline phosphatase, gammaGT) and neutropenia (Blood count).  **3. Predictors of efficacy of Anakinra :**  The association between several of clinical parameters at inclusion (including level of oxygen requirement, respiratory rate, temperature…), biological parameters (including, CRP, ferritin, LDH, lymphocyte count, eosinophil count, Ddimers, platelet count, polymorphonuclear count...), and cytokines profile analysis with the primary end point will be explored. |
| Participants | **Inclusion criteria :**   - Male or female≥ 18 years of age - Written informed consent of the patient or a proxy - Ability for participant to comply with the requirements of the study - Hospitalized patient with COVID-19 defined as   - **Positive SARS-CoV2 RT-PCR**   - Or **typical COVID-19 Radiographic infiltrates on the CT scan** (peripheral ground glass without lung cavitation, lymphadenopathy, or pulmonary nodules) **other non COVID-19 diagnosis ruled out**. - Patient with respiratory symptoms and requirement of oxygen therapy as defined :   - **Oxygen therapy >= 4L/min to maintain Sp02>92% and respiratory rate >=24/min.**   - **Or patients under oxygen >= 1L/min and presenting worsening of oxygen requirement defined by an increase of oxygen therapy >= 2L/min to maintain Sp02>92%.** - Inflammatory component **C**-**Reactive Protein ≥ 50mg/L.** - Patients within the first 20 days from the onset of the first COVID-19 symptoms - Probabilistic antibiotics therapy according to local practice   **Non-inclusion criteria :**   - Respiratory failure related to other cause than COVID-19 - **Patients requiring mechanical ventilation at inclusion or requiring oxygen therapy equal or more than 11 liters per min to maintain Sp02>92%** - **Infectious diseases such as severe bacterial infections, aspergillosis,  HIV, active HCV, active HBV, active tuberculosis** - Contra indication to anti-IL1 receptor - Known hypersensitivity to Anakinra - Absolute neutrophil count (ANC)<  1500/mm3 - Liver cirrhosis score de Child-Pugh class C - Live or attenuated vaccine in the past 8 weeks - Pregnant or breast-feeding women - Patients with either legally protected status or who have been deprived of their freedom - Patient included in other interventional therapeutic research (e.g. = concurrent participation in French CoVID-19 is accepted) - Patients who have received previous treatment by anti-IL6R, anti-IL-6, anti-IL1R, anti-IL1 or anti-TNFα within 21 days preceding inclusion - Absence of Health Insurance - Existence of any life-threatening co-morbidity or any other medical condition which,in the opinion of the investigator, makes the patient unsuitable for inclusion. |
| Interventions | **The experimental group** will receive **Anakinra** plus **optimized Standard of Care**.  The patients will receive Intraveinous injection (IV) of **Anakinra 400mg/day (100mg IV every 6 hours) at Day 1, 2 and 3**. From Day 4 to Day 10, the patient will receive IV injection of Anakinra **200mg/day (100mg every 12 hours). The total duration of Anakinra is 10 Days** |
|  | **The control group** will receive **optimized standard of care** alone, including all treatments authorized for COVID-19 by the French Health Ministry and/or the center COVID-19 therapeutic committees at inclusion and during the follow-up. |
| Participant Timeline | **Day 0** is the day the patient is included and started the treatment **(V1)**. Patients will be followed daily during daily during hospitalization and **study visits will be performed at Day 3 (V2), Day 10 (V3), Day 14 (V4) and Day 28 (V5).**  **If the patient is discharged from the hospital** **at V4 or V5**, a blood test will be prescribed and phone visits will be organized to record status, concomitant medication and AEs/AES. |
| randomization and blinding | Randomization :  Randomization will be performed at the end of the inclusion visit when all the eligibility criteria are checked.  Participants will be randomly assigned to receive either Anakinra plus optimized standard of care (oSOC) or oSOC alone in a 1:1 ratio as per a computer (SAS based) generated randomisation schedule. Randomization will be stratified on the **baseline CRP concentration** (<150 vs. >=150 mg/L), baseline **requirement of oxygen therapy** to maintain Sp02 92% (3-6 liters per min vs. 7-10 liters per min) and **corticosteroid therapy at** baseline (>=0.5 mg/kg/day) (yes vs.no) using permuted blocks of random sizes.  Blinding :  Due to feasibility issues in the current emergency context, this trial is open label without placebo arm. |
| Sample size | **240 patients** (120 per group) with an interim analysis on the first 120 patients (60 per group). |
| Expected Duration of the study | Expected enrolment duration : 4 months maximum (**expected 2 months**)  Duration of treatment: 10 days  Duration of the study for a participant: **28 days**  Total duration of the study: **4 months and 28** days maximum (expected 3 months*)* |
| Data and Safety Monitoring Board | The study may be stopped definitely or temporarily at any time by the sponsor on the basis of information provided by the Data and Safety Monitoring Board (DSMB). The **DSMB** will meet before the trial initiation, at **one interim analysis** (after the inclusion of 120 patients) for efficacy analysis and e**ach 60 patients (60, 120, 180), for safety concerns**. |
| Collection of biological samples | Use of biological samples in the context of this research with preservation and subsequent use of this collection.  Duration of preservation beyond the study: until all of samples have been used  Stored at “Centre de Recherche Biologique” (CRB) of CHRU of Tours |
| Feasibility | **47 COVID-19 centers** will participate to the study. COVID referent centers, all over French territory will allow a **nationa**l and **rapid recruitment** to conduct this trial. **From 20 to 90 COVID-19 patients are currently hospitalized in each of the participating centers**. The vast majority of French University Hospitals (CHU) and one military hospital « Hôpital d'Instruction des Armées » HIA agreed to participate to this trial. The vast majority of the « Hôpitaux Universitaires du Grand Ouest » HUGO network has been solicited. |
| Expected Results | So far, COVID-19 has infected more than 1.3 million of peoples all around the world and caused death of more than 70 000 persons. Up to 20% of hospitalized patients need an admission in intensive care medicine to receive ventilation support and the vast majority experienced acute respiratory distress syndrome (ARDS). **To date, there is no efficient therapeutics to prevent or treat COVID-19 related respiratory failure**. In France, as in others countries, number of patients infected and death are exponentially growing. **Identifying a potentially efficient drug is a major concern and a public health emergency.** In addition, some French Hospitals, in particular in the eastern part of the country, are reaching a saturation point and can no longer admit new patients in Intensive Care Units. Therefore, identifying an efficient drug to limit patient’s admission in ICU and to limit COVID-19 induced mortality is crucial. Favorable results could lead to new evidence-based recommendations for care of patients affected by COVID-19. |

**LIST OF ABBREVIATIONS**

| AE | Adverse Event |
| --- | --- |
| ANSM | Agence Nationale de Sécurité des Médicaments et des produits de santé, French National Agency for Medicines and Health Products safety |
| AMM | Autorisation de Mise sur le Marché  Marketing authorization |
| CPP | Comité de Protection des Personnes  Protection of individuals committee / Ethics committee |
| CNIL | Commission Nationale de l'Informatique et des Libertés  French National Commission for Information Technology and Civil Liberties |
| CRA | Clinical Research Assistant |
| DSMB | Data Safety Monitoring Board |
| DSUR | Development Safety Update Report |
| e-CRF | Electronic Case Report Form |
| GCP | Good Clinical Practices |
| ICH | International Conference on Harmonisation |
| IP | Investigational Product |
| INSERM | Institut National de la Santé et de la Recherche Médicale  French National Health and Medical Research Institute |
| ITT | Intention to Treat |
| MR | Méthodologie de référence  Reference methodology |
| oSOC | Optimized Standard Of Care |
| REC | Research Ethics Committee |
| SAE | Serious Adverse Event |
| SPC | Summary of Product Characteristics |
| SOP | Standard Operating Procedures |
| SUSAR | Suspected Unexpected Serious Adverse Reaction |
|  |  |

**TABLE OF CONTENTS**

[Randomization : 11](#__RefHeading___Toc37362848)

[Blinding : 11](#__RefHeading___Toc37362849)

[1. Background and rationale 18](#__RefHeading___Toc37362850)

[1.1. Background 18](#__RefHeading___Toc37362851)

[1.2. Study rationale 19](#__RefHeading___Toc37362852)

[2. Objectives 19](#__RefHeading___Toc37362853)

[2.1. Primary objective of the study 19](#__RefHeading___Toc37362854)

[2.2. Secondary objectives of the study 19](#__RefHeading___Toc37362855)

[3. Study design 20](#__RefHeading___Toc37362856)

[4. Outcomes 20](#__RefHeading___Toc37362857)

[4.1. Primary Outcome 20](#__RefHeading___Toc37362858)

[4.2. Secondary Outcomes 20](#__RefHeading___Toc37362859)

[5. Study setting 21](#__RefHeading___Toc37362860)

[6. Participants 21](#__RefHeading___Toc37362861)

[6.1. Inclusion criteria 21](#__RefHeading___Toc37362862)

[6.2. Non-inclusion criteria 22](#__RefHeading___Toc37362863)

[6.3. Exclusion period for the participant’s inclusion in another study if suitable 23](#__RefHeading___Toc37362864)

[7. Interventions, 23](#__RefHeading___Toc37362865)

[Anakinra is the IP (Investigational Product) in this study. 23](#__RefHeading___Toc37362866)

[7.1. Experimental group 23](#__RefHeading___Toc37362867)

[7.1.1. Drug characteristics 24](#__RefHeading___Toc37362868)

[7.1.2. Administration 24](#__RefHeading___Toc37362870)

[7.1.3. Contraindications 24](#__RefHeading___Toc37362874)

[7.2. Control group 24](#__RefHeading___Toc37362875)

[7.3. Investigational product management 24](#__RefHeading___Toc37362876)

[7.3.1. Supply of products 24](#__RefHeading___Toc37362877)

[7.3.2. Packaging and labelling 25](#__RefHeading___Toc37362878)

[7.3.3. Storage conditions 25](#__RefHeading___Toc37362879)

[7.3.4. Drug accountability 25](#__RefHeading___Toc37362880)

[7.3.5. Return and destruction of unused products 25](#__RefHeading___Toc37362881)

[7.4. Intervention modifications during the study 25](#__RefHeading___Toc37362882)

[7.5. Intervention adherence 25](#__RefHeading___Toc37362883)

[7.6. Intervention concomitant care 25](#__RefHeading___Toc37362884)

[7.6.1. Authorized concomitant care 25](#__RefHeading___Toc37362885)

[7.6.2. Prohibited concomitant care 26](#__RefHeading___Toc37362886)

[8. Participant timeline 26](#__RefHeading___Toc37362887)

[8.1. Participant selection and recruitment 26](#__RefHeading___Toc37362888)

[8.2. Inclusion and baseline assessment 26](#__RefHeading___Toc37362889)

[8.3. Follow-up assessments and visit 27](#__RefHeading___Toc37362891)

[8.3.1. **Visit 1** 27](#__RefHeading___Toc37362895)

[8.3.2. **Visit**  28](#__RefHeading___Toc37362896)

[8.3.3. **Visit 3** 29](#__RefHeading___Toc37362907)

[8.3.4. **Visit 4** 29](#__RefHeading___Toc37362909)

[8.3.5. **Visit 5** 30](#__RefHeading___Toc37362911)

[8.4. Patients care after the end of the participation 32](#__RefHeading___Toc37362913)

[8.5. Discontinuation and withdrawal 32](#__RefHeading___Toc37362915)

[8.6. Discontinuation of the study 33](#__RefHeading___Toc37362916)

[8.7. Duration of the study 33](#__RefHeading___Toc37362917)

[9. Randomization 33](#__RefHeading___Toc37362918)

[9.1. Sequence generation 33](#__RefHeading___Toc37362919)

[9.2. Implementation 34](#__RefHeading___Toc37362920)

[9.3. Allocation concealment mechanism 34](#__RefHeading___Toc37362921)

[10. Blinding 34](#__RefHeading___Toc37362922)

[11. Other strategies to reduce bias 34](#__RefHeading___Toc37362923)

[12. Data handling 34](#__RefHeading___Toc37362924)

[12.1. Data collection 34](#__RefHeading___Toc37362925)

[12.1.1. Access to data34](#__RefHeading___Toc37362926)

[12.1.2. Source data and source document34](#__RefHeading___Toc37362927)

[12.2. Data collection tool 35](#__RefHeading___Toc37362928)

[12.3. Confidentiality of data 35](#__RefHeading___Toc37362929)

[12.4. Data management procedures 35](#__RefHeading___Toc37362930)

[12.5. Data validation 36](#__RefHeading___Toc37362931)

[12.6. Security and archival of data 36](#__RefHeading___Toc37362932)

[13. Statistical considerations 36](#__RefHeading___Toc37362933)

[13.1. General principles of study analysis 36](#__RefHeading___Toc37362934)

[13.2. Analysis population definition 36](#__RefHeading___Toc37362935)

[13.3. Baseline characteristics 36](#__RefHeading___Toc37362936)

[13.4. Statistical analysis of the primary outcome 37](#__RefHeading___Toc37362937)

[13.5. Statistical analysis of secondary outcomes 37](#__RefHeading___Toc37362938)

[13.6. Additional analyses 38](#__RefHeading___Toc37362939)

[13.7. Interim analyses 38](#__RefHeading___Toc37362940)

[13.8. Sample size 39](#__RefHeading___Toc37362941)

[14. Project feasibility 39](#__RefHeading___Toc37362942)

[15. Expected results/benefits 39](#__RefHeading___Toc37362943)

[16. Evaluation of security 40](#__RefHeading___Toc37362944)

[16.1. Description of safety evaluation parameters 40](#__RefHeading___Toc37362945)

[16.2. Procedures and timing for the measurement, collection and analysis of the safety evaluation parameters 41](#__RefHeading___Toc37362950)

[16.3. Procedures in place for the documentation and the reporting of serious adverse events 41](#__RefHeading___Toc37362951)

[16.3.1. Investigator's responsibilities](#__RefHeading___Toc37362952)

[16.3.1.1. Reporting of serious adverse events 41](#__RefHeading___Toc37362953)

[16.3.1.1.1. Information to be reported to the sponsor41](#__RefHeading___Toc37362954)

[16.3.1.1.2. Procedure for SAE reporting to the sponsor42](#__RefHeading___Toc37362970)

[16.3.1.1.3. Time limit for SAE reporting to the sponsor42](#__RefHeading___Toc37362973)

[16.3.1.1.4. Reporting period of SAE to the sponsor42](#__RefHeading___Toc37362976)

[16.3.1.2. Reporting of non-serious adverse events 42](#__RefHeading___Toc37362979)

[16.3.1.3. Specificities of the protocol 43](#__RefHeading___Toc37362981)

[16.3.2. Sponsor's responsibilities43](#__RefHeading___Toc37362982)

[16.3.2.1. Analysis of serious adverse events 43](#__RefHeading___Toc37362983)

[16.3.2.2. Declaration of suspected unexpected serious adverse reactions 43](#__RefHeading___Toc37362984)

[16.3.2.3. Transmission of annual safety reports DSUR (Development Safety Update Report) 44](#__RefHeading___Toc37362985)

[16.3.2.4. Declaration of other safety data 44](#__RefHeading___Toc37362986)

[16.3.2.5. Data and Safety Monitoring Board (DSMB) 44](#__RefHeading___Toc37362987)

[16.3.3. In utero exposure 45](#__RefHeading___Toc37362988)

[17. Practical issues on study sites 45](#__RefHeading___Toc37362995)

[18. Quality control – Monitoring visits 46](#__RefHeading___Toc37362996)

[19. Audit and inspection 46](#__RefHeading___Toc37362997)

[20. Storage of documents and data at the end of the study 46](#__RefHeading___Toc37362998)

[20.1. By the investigators: 46](#__RefHeading___Toc37362999)

[20.2. By the sponsor: 46](#__RefHeading___Toc37363000)

[21. Administrative, ethical and regulatory considerations 47](#__RefHeading___Toc37363001)

[21.1. Information and consent forms 47](#__RefHeading___Toc37363002)

[21.2. CNIL 47](#__RefHeading___Toc37363003)

[21.3. Research ethics committee 47](#__RefHeading___Toc37363004)

[21.4. Regulatory authorities 47](#__RefHeading___Toc37363005)

[21.5. Biological collection 48](#__RefHeading___Toc37363006)

[21.6. Protocol amendments 48](#__RefHeading___Toc37363007)

[21.7. Registration 48](#__RefHeading___Toc37363008)

[21.8. Insurance 48](#__RefHeading___Toc37363009)

[22. Dissemination policy 48](#__RefHeading___Toc37363010)

[22.1. Authorship 48](#__RefHeading___Toc37363011)

[22.2. Communication of the results to participants 49](#__RefHeading___Toc37363012)

[22.3. Study data sharing statement 49](#__RefHeading___Toc37363013)

[23. Financial data 49](#__RefHeading___Toc37363014)

[23.1. Budget of the study 49](#__RefHeading___Toc37363015)

[23.2. Compensation for participants 49](#__RefHeading___Toc37363016)

[REFERENCES 50](#__RefHeading___Toc37363017)

[APPENDIX 52](#__RefHeading___Toc37363018)

[Appendix 1. List of study sites 52](#__RefHeading___Toc37363019)

[Appendix 2. Safety evaluation terminology 57](#__RefHeading___Toc37363020)

[Appendix 3. Severity evaluation of non-serious adverse events 59](#__RefHeading___Toc37363021)

[Appendix 4. Causal relationship evaluation 60](#__RefHeading___Toc37363022)

1. Background and rationale
   1. Background

Severe acute respiratory syndrome coronavirus 2 (SARS-Cov-2) is an emergent coronavirus, first reported in Wuhan, China 2019. The disease is named Corona Virus Disease-19 (COVID-19). So far, the virus has infected more than 1.3 million peoples all around the world and caused the death of more than 74 000 patients.

Up to 20% of hospitalized patients need an admission in intensive care medicine to receive ventilation support and the vast majority experienced acute respiratory distress syndrome (ARDS) [1-2]. To date, there is no efficient therapeutics to prevent or treat COVID-19 related respiratory failure. Identifying a drug is a major concern and a public health emergency.

**The pathogenesis of COVID-19 encompasses a cytokine storm which includes pro-inflammatory interleukins (IL-1β, IL-6) and tumour necrosis factor (TNF-α)** [3]. Several studies suggest that patients who develop severe respiratory forms of COVID-19 have a deleterious pulmonary and systemic inflammatory cascade. **Predictors of mortality from a recent retrospective, multicentre study of 150 confirmed COVID-19 cases in Wuhan, China, included elevated ferritin and elevated C-Reactive Protein (CRP) (mean 126 mg/L in non survivors vs. 34 mg/L in survivors) suggesting that mortality might be due to virally driven hyperacute inflammation** [4]. It is well known that circulating CRP is produced by hepatocytes under the regulatory control from circulating cytokines, in particular IL-6 and IL1β.

Monocytes are key mediators of early host responses to microbial infections [6]. The steady state recruitment of blood monocytes into peripheral tissues and local differentiation of these cells *in situ* is accelerated during infection, allowing the rapid replenishment of macrophage and dendritic cell populations in infected tissues.

Growing evidence implicates excessive monocyte/macrophage activation and associated cytokine storm with the pathophysiology of severe SARS-CoV-2 disease related complications. Zhou and colleagues recently reported on the presence of a significantly higher percentage of CD14+ CD16+ inflammatory monocytes in the peripheral blood of COVID-19 patients compared to normal healthy controls [7]. They also reported that the percentage of CD14+ CD16+ monocytes was much higher in severe pulmonary syndrome patients from ICU. Moreover, morphological and functional changes, which are more pronounced in patients requiring prolonged hospitalization and ICU admission, were rapidly observed in these cells [8]. Lastly, scRNA screening performed on lung bronchoalveolar lavage fluid (BALF) cells from COVID-19 patients revealed that inflammatory monocytes are recruited from circulation to the lung to fuel the inflammation during severe diseases and replenish tissue myeloid cell compartment [9].

Unlike tissue-resident cells such as macrophages, circulating monocytes monocyte-derived cells exhibit extremely rapid and robust responses to infections by producing produce large amounts of pro-inflammatory cytokines including IL-1α/β, TNFα and IL-6. Although IL-1β exerts potent host protective effects during infection, excess production of this cytokine is also associated with septic shock, deregulated inflammation, and autoimmune diseases [10].

- 1. Study rationale

**Therefore, we suggest that IL-1β is a potentially therapeutic target to stop the inflammation process and to treat patient at risk for developing severe ARDS related to COVID-19.** As a recombinant anti-human IL-1 receptor treatment, Anakinra can specifically bind to IL-1R and inhibit signal transduction. **For a decade, Anakinra is mainly used to treat patients with rheumatoid arthritis or auto-inflammatory diseases and its safety profile is favourable in such indication.**

Moreover, **subgroup analysis of data from a phase III randomized controlled trial of IL-1 blockade (Anakinra) in sepsis, showed significant survival benefit in patients with hyperacute inflammation, without increased adverse events as compared to placebo**. In this trial, the 28-day survival rate in patients with hyperacute inflammation was 65.4% with Anakinra vs. 35.3% with placebo, HR = 0.28 (95% CI 0.11–0.71, p = 0.0071) [5].

Several multicentre, randomized controlled trials of tocilizumab (IL-6 receptor blockade) and sarilumab (IL-6 neutralisation) are ongoing in many countries for COVID-19 patients.

We, therefore, aimed to study the efficacy and safety of Anakinra plus optimized standard of care compared to optimized standard of care alone, in patients with COVID-19 with aggravating respiratory symptoms and inflammatory component in a multicenter randomized clinical trial.

1. Objectives
   1. Primary objective of the study

The main objective of the ANACONDA trial is to **assess the efficacy of Anakinra + optimized Standard of Care (oSOC) as compared to oSOC alone** on the condition of patients with COVID-19 infection and worsening respiratory symptoms. **Success defined as patient alive and free of invasive mechanical ventilation (IMV) and free of Extracorporeal Membrane Oxygenation (ECMO) at Day** 14.

- 1. Secondary objectives of the study

1. **To assess the efficacy of Anakinra + oSOC as compared to oSOC alone on:**

- Treatment success (same definition than the primary outcome) up to Day 28
- Patient’s condition as defined by the OMS 7 point scale up to Day 28
- Patient’s mortality up to day 28
- Patient’s admission in ICU
- Pulmonary function (need for ventilator support, SP02, PaO2/FiO2) up to Day 28
- Inflammation parameters up to Day 28

1. **To evaluate the safety profile of Anakinra**
2. **To identify predictors of efficacy of Anakinra.**
3. Study design

ANACONDA study is a French multicentre, open-label, randomized, controlled superiority trial comparing the administration of optimized standard of care and Anakinra versus optimized standard of care alone in patients hospitalized in a medical unit with COVID-19.

After consent, eligible patients will be randomly allocated with a 1:1 ratio to either Anakinra plus optimized standard of care or optimized standard of care alone. **Randomization** will be stratified on the baseline **CRP value** (<150 vs. >=150 mg/l), **baseline requirement of oxygen therapy to maintain Sp02 over 92%** (3-6 liters per min vs. 7-10 liters per min), and **corticosteroid** therapy at baseline (< vs. >=0.5mg/kg/day prednisone) (yes vs no). Patients will be followed up to day 28. Baseline measurements will include clinical, radiological and biological measurements. Patients will be followed daily during daily during hospitalization and study **visits will be performed at Day 0, 3, Day 10, Day 14 and Day 28.**

Due to feasibility concern in the current emergency circumstances, the trial is open labelled (no placebo arm) but the primary outcome is objective thus preventing any detection bias in the trial results.

We plan to recruit **240 patients**, **120 patients per group**. **In order to allow an early stopping of the trial for efficacy, an interim analysis will be performed after the primary outcome has been obtained for the first 120 patients.** We will use a Pocock’s approach with a p-value boundary of 0.0294 for both the interim and final analysis [11]. Results of the interim analysis will be reviewed by a data safety and monitoring board (DSMB) who will provide a recommendation on whether the trial should be carry on or not. **The DSMB will also review the safety data on a regular basis (every 60 patients included).**

1. Outcomes
   1. Primary Outcome

The primary endpoint is treatment success at **Day 14**, defined as a **patient alive** and **not requiring any of the following: Invasive mechanical ventilation (IMV) or Extracorporeal membrane oxygenation (ECMO).**

- 1. Secondary Outcomes

**1. Efficacy outcomes :**

- Treatment success (same definition as the Primary outcome) at Day 3, Day 10 and Day 28
- OMS progression scale (on a 7-point ordinal scale): at Day 3, Day 10, Day 14 and Day 28

1. Not hospitalized, no limitations on activities

2. Not hospitalized, limitation on activities;

3. Hospitalized, not requiring supplemental oxygen;

4. Hospitalized, requiring supplemental oxygen;

5. Hospitalized, on non-invasive ventilation or high flow oxygen devices;

6. Hospitalized, on invasive mechanical ventilation or ECMO;

7. Death.

- Overall survival at Day 3, Day 10, Day 14 and Day 28
- Time to ICU admission
- Time to ventilatory support (ECMO, invasive mechanical ventilation, non-invasive ventilation, high flow oxygen therapy)
- Change in NEWs [12] score from baseline to Day 3, Day 10, Day 14 and Day 28
- Change in HScore [13] from baseline to Day 3, Day 10, Day 14 and Day 28
- Change in inflammatory parameters (CRP, ferritin, D-dimer, fibrinogen, lymphocytes count, platelet count) from baseline to Day 3, Day 10, Day 14 and Day 28
- Hospital length of stay: Time from inclusion to hospital discharge

For those admitted in ICU:

- Need for Vasopressors (yes or no)
- If IMV, Evolution of SpO2/FIO2
- If IMV, Evolution of PaO2/FiO2 ratio
- ICU length of stay: Time from admission in ICU to ICU discharge

Secondary outcomes of the trial will be updated according to the COS for COVID-19 but primary outcome will not change.

**2. The safety outcomes :**

Occurrence of serious adverse events during the study, including infection (bacterial, parasitic, mycotic and viral infection), septic shock, Ankanira hypersensitivity, hepatic damages (SGOT/SGPT, alkaline phosphatase, gammaGT) and neutropenia (Blood count).

**3. Predictors of efficacy of Anakinra :**

The association between several clinical parameters at inclusion (including level of oxygen requirement, respiratory rate, temperature…), biological parameters (including, CRP, ferritin, LDH, lymphocyte count, eosinophil count, Ddimers, platelet count, polymorphonuclear count...), and cytokines profile analysis (ancillary analysis, optional) with the primary end point will be explored.

1. Study setting

The ANACONDA trial will take place in **47 French COVIDS-19 centers**.

The list of study sites is provided in **Appendix 1.**

1. Participants
   1. Inclusion criteria

- Male or female≥ 18 years of age
- Participants covered by or entitled to social security
- Written informed consent of the patient or a proxy
- Ability for participant to comply with the requirements of the study
- Hospitalized patient
- Patient with COVID-19 defined as:
  - Positive SARS-CoV2 RT-PCR
  - Or typical COVID-19 Radiographic infiltrates on the CT scan (peripheral ground glass without lung cavitation, lymphadenopathy, or pulmonary nodules) others non COVID-19 diagnosis ruled out.
- Patient with respiratory symptoms and requirement of oxygen therapy as defined :
  - Oxygen therapy >= 4L/min to maintain Sp02>92% and respiratory rate >=24/min.
  - Or patients under oxygen >= 1L/min and presenting worsening of oxygen requirement defined by an increase of oxygen therapy >= 2L/min to maintain Sp02>92%.
- Inflammatory component Reactive C-Protein ≥ 50mg/L.
- Patients within the first 20 days from the onset of the first COVID-19 symptoms.
- Probabilistic antibiotics therapy according to local practice
  1. Non-inclusion criteria
- Respiratory failure related to other cause than COVID-19
- Patients requiring mechanical ventilation at inclusion or requiring oxygen therapy equal or more than 11 liters per min to maintain Sp02>92%
- Infectious diseases such as severe bacterial infections, aspergillosis,  HIV, active HCV, active HBV, active tuberculosis
- Contra indication to anti-IL1 receptor
- Known hypersensitivity to Anakinra
- Absolute neutrophil count (ANC) < 1500/mm3
- Liver cirrhosis Child-Pugh score C
- Live or attenuated vaccine in the past 8 weeks
- Pregnant or breast-feeding women
- Patients with either legally protected status or who have been deprived of their freedom
- Patient included in other interventional therapeutic research (e.g. = concurrent participation in French CoVID-19 is accepted)
- Patients who have received previous treatment by anti-IL6R, anti-IL-6, anti-IL1R, anti-IL1 or anti-TNFα within 21 days preceding inclusion
- Absence of Health Insurance
- Existence of any life-threatening co-morbidity or any other medical condition which in the opinion of the investigator makes the patient unsuitable for inclusion.
  1. Exclusion period for the participant’s inclusion in another study if suitable

Included participants will not be allowed to be involved in another pharmacological study during the entire follow-up period. But participation in an observational study will be allowed (e.g French COVID 19 cohort). No exclusion period after the end of the trial participation.

1. Interventions,

Anakinra (Kineret®) is the IP (Investigational Product) in this study.

- 1. Experimental group

In the **experimental group**, patients will receive **Anakinra** plus **optimized Standard of Care**.

- **Optimized Standard of Care**

The Anakinra group will also receive the «optimized standard of care», **», included all treatments authorized for COVID-19 by the French Health Ministry and/or the center COVID-19 therapeutic committees at inclusion and during the follow-up**.

- **Anakinra**

The patients will receive Intraveinous injection (IV) of **Anakinra 400mg/day (100mg IV every 6 hours) at Day 1, 2 and 3**. From Day 4 to Day 10, the patient will receive IV injection of Anakinra **200mg/day (100mg every 12 hours). The total duration of Anakinra is 10 Days**

In patients with severe renal impairment (CLcr <30 mL/minute) or end-stage renal disease, including those on dialysis, Anakinra will be administered every 2 days. In the geriatric population (≥ 65 years old) no dose adjustment is necessary. In patients with hepatic insufficiency, no dose adjustment is necessary for patients with moderate hepatic impairment (Child-Pugh Class A or B score).

**Dose selection rationale for Anakinra :**

Anakinra is approved for the chronic treatment of a number of inflammatory diseases as a subcutaneous treatment (at doses of **100 mg/day** or in weight-based doses of up to **8 mg/kg/day**). The IV administration of anakinra has been studied in clinical trials in healthy volunteers and in critically ill patients with sepsis and hyperacute inflammation at varying IV doses up to **3500 mg/day** over 72 hours: e.g., 2 mg/kg/hour**, 20 mg/kg/day** (<40 kg) and 916 mg/day (> 40kg), bolus of 100 mg followed by infusion of 2 mg/kg/hour. No safety concerns emerged in these studies [15-18].

So far, **Anakinra efficacy and dose to reach efficacy is not known in COVID-19 patients.** In order to select the appropriate dose of Anakinra, for the current trial, **we have set up a Scientific Committee** composed of French **experts** who prescribe Anakinra in inflammatory diseases: **Prof. Achille AOUBA,** Internal Medicine Department, Caen Hospital and **Prof. Arsène MEKINIAN**, Internal Medicine Department, Hospital Saint Antoine. The Scientific Committee has also solicited French physicians treating COVID-19 patients with Anakinra (off label). Thus, **based on the Scientific Committee** advice and **considering benefits/risks balance**, Anakinra will be administered **as described above**

- - 1. Drug characteristics

Anakinra (Kineret®) Swedish Orphan Biovitrum (SOBI) is an authorized medicine in Europe since 08/03/2002. Anakinra is indicated in adults for: rheumatoid arthritis (in combination with methotrexate), CAPS (Cryopyrin Associated Periodic Syndrome), Muckle-Wells syndrome (MWS), familial cold auto inflammatory syndrome (FCAS), in adults, adolescents, children and infants aged 8 months for Still’s disease, including Systemic Juvenile Idiopathic Arthritis (SJIA) and Adult-Onset Still’s Disease (AOSD). Kineret blocks the biological activity of IL-1 by binding to the interleukin-1 type 1 receptor, expressed in a wide variety of cells. ATC code: L04AC03.In this study, treatment by Kineret® for injection 100mg solution injects will be used for participants. The experimental treatment is anakinra 100mg/0.67 mL solution for injection in pre-filled syringe (Swedish Orphan Biovitrum SE-112 76 Stockholm - Sweden). Anakinra must be stored at refrigerated conditions at 2-8 °C (36°-46°F) in a secure area at the study sites. Further instructions for handling and storage of the IMP anakinra are available in the IMP manual.

- - 1. Administration

The patients will receive Intraveinous injection (IV) of Anakinra 400mg/day (100mg IV every6 hours) during 3 days. Then, the patient will receive IV injection of Anakinra 200mg/day (100mg every 12 hours) during 7 days. The total duration of Anakinra is 10 Days. Before administration the full content of the prefilled, single use syringe (Anakinra 100 mg) will be diluted in 100 mL saline. The IV administration of Anakinra has to occur immediately after the preparation over an infusion period of 60 minutes. Full instructions for the preparation of Anakinra are available in the IMP manual.

- - 1. Contraindications to Anakinra
- Known hypersensitivity to Anakinra or to any exciptients
- Absolute neutrophil count (ANC) < 1500/mm3
- Liver cirrhosis Child-Pugh C score
- Live or attenuated vaccine in the past 8 weeks
- Pregnant or breast-feeding women
  1. Control group

The **control group** will receive «optimized standard of care» alone, including all treatments authorized for COVID-19 by the French Health Ministry and/or the center COVID-19 therapeutic committees at inclusion and during the follow-up.

- 1. Investigational product management
     1. Supply of products

IP treatment will be distributed to each investigational site by the Hospital pharmacy of Tours.

- - 1. Packaging and labelling

The Investigational product will be used in its commercialized form.

The treatments will be labelled in accordance to clinical trial regulations by the Hospital pharmacy of Tours.

- - 1. Storage conditions

Treatments will be supplied to the pharmacist of each investigational site, who will be in charge of the traceability and the storage.

The study experimental treatments will be stored in accordance with regulations, in a different place to the other drugs of the pharmacy, with restricted and locked access and according to the storage conditions recommended by the manufacturer. The products must be stored at controlled refrigerated temperature (2 to 8°C) away from light. Temperature logs should be kept updated by the investigator or the pharmacist in order to document adequate storage during the course of the study.

- - 1. Drug accountability

The investigator is responsible for ensuring that all study drugs received at the site are inventoried and accounted throughout the study. The local pharmacy will be in charge of the accountability of the study treatment. The dispensing of a study drug to the participant, and the return of a study drug (unused drug), must be documented on the drug accountability form. An unused study drug and/or a study drug returned by the clinical unit must be available for verification by the sponsor's site monitor during on-site monitoring visits.

- - 1. Return and destruction of unused products

All remaining used and unused IPs will be collected and destroyed at the end of the study. IPs will be kept at the local pharmacy until the monitoring visit by the CRA. Then they will be destroyed on site after a written agreement by the CRA.

- 1. Intervention modifications during the study

Intervention modifications are described in paragraph 7.1.2

- 1. Intervention adherence

Participants will be asked to keep a participant diary noting information concerning adherence and safety data.

- 1. Intervention concomitant care
     1. Authorized concomitant care

Analgesic treatment, transfusion of blood products, electrolyte and glucose infusions, IV, parenteral nutrition, inotropic support, antibiotics, anti-fungal and anti-viral treatments, ultrafiltration or hemodialysis, as well as general supportive care are allowed. Other therapy considered necessary for the patient’s welfare may be given at the discretion of the Investigator. All relevant concomitant therapy, as defined by the Investigator, will be recorded in the CRF.

- - 1. Prohibited concomitant care

No interaction study between Anakinra and other drugs has been performed. During clinical trial, no interaction between Anakinra and other drugs (glucocorticoids and background treatments for rheumatoid arthritis (DMARD)) has been observed. **Concomitant use of IL-6 inhibitors** (e.g., tocilizumab)**, non-anakinra IL-1 inhibitors** (e.g, canakinumab), **TNF inhibitors, JAK inhibitors is not allowed**. Lived or attenued vaccination during the study are not authorized.

1. Participant timeline
   1. Participant selection and recruitment

**47 centers** will participate in the study. COVID referent centers, all over French territory will allow a **nationa**l and **rapid recruitment** to conduct this trial. F**rom 20 to 90 COVID-19 patients are currently hospitalized in each of the participating centers**. The vast majority of French University Hospitals (CHU) and one military hospital « Hôpital d'Instruction des Armées » HIA agreed to participate to this trial. The vast majority of « Hôpitaux Universitaires du Grand Ouest » HUGO network participated to this study. All patients with a positive COVID-19 diagnosis will be considered for inclusions. Patients will be selected for recruitment in the care units where are hospitalized COVID-19 patients. Some inclusion and exclusion criteria will be first checked during a routine/daily medical visit. At this time, the physician will present the study to the patient and give him/her an information letter.

- 1. Inclusion and baseline assessment

Patients will be included in the study after obtaining all the information necessary to understand the study. The investigator will explain to the patient the purpose of the study, its methodology, the expected benefits and risks of the treatments. The written and informed consent of the patient must be dated and signed both by the patient and the investigator before any further study assessment. The patient will receive a copy of signed written consent and information letter. After the signature of the written and informed consent, the procedures for study will begin. To check biological criteria, a blood test will be performed if the last results available are dated more than 24 hours, including urine or blood pregnancy test for women of childbearing potential.

Specific Consent for participant’s representative in case the patient is not able to give his/her consent will be proposed. If there is no representative present, the inclusion is carried out by the investigator as part of an inclusion procedure. As soon as possible, the patient or a patient representative is informed and the collection of his/her oral consent will be documented with the need to collect the confirmation of the relative's/family's written consent as soon as possible and by any means (mail, e-mail, etc.).

In the two cases, the patient is informed as soon as possible, if he or she recovers his or her capacity to consent, and his or her consent should be obtained for the eventual continuation of the study until the end of his or her participation in the study. Inclusion as part of an emergency procedure is indicated where appropriate by specifying the modalities (inclusion by a patient representative or inclusion without consent if no representative is present at the time of inclusion).

**After verification of all the inclusion and exclusion criteria**, the following procedures will then be performed:

- Collection of data: age, gender, comorbidities (diabetes, tobacco, hypertension, …) previous treatments (biotherapy, NSAI, or other significate treatment), concomitants treatments ongoing
- Record weight and height
- Perform urine or blood pregnancy for women of childbearing potential. If the test is positive the patient should not be randomized and must be withdrawn from the study before randomization.

**The duration of screening period must not exceed 24 hours before randomization.**

- 1. Follow-up assessments and visit

The Day 0 will correspond to the day of randomization; the start of treatment for the Anakinra group should start as soon as possible after randomization. The randomization will correspond to the Day 0 (H-0) for the 2 groups.

- - 1. **Visit 1** (Day 0-H0)

During the **Visit 1**, the following procedures will be performed:

- Physical exam including research of hepatomegaly, splenomegaly.
- Collection of vital parameters: Temperature, Blood Pressure, pulse, respiratory rate, conscious, oxygen flow and saturation. For the Anakinra group, the vital parameters must be collected just before administration of treatment.
- Perform blood test: blood count, platelets, C-Reactive Protein, creatinine + clearance (MDRD), ferritin, SGOT/SGPT, alkalines phosphatases, gammaGT, triglycerides, LDH, CPK, , fibrinogen, troponin, BNP, D-Dimers.
- Perform serum and plasma bank sampling if possible in the center and after written authorization of the patient.
- If the patient is allocated in the interventional group, the first intravenous infusion of IV injection Anakinra 100mg should be administered as soon as possible after the randomization.
- For the Anakinra group, the treatment will be administered 4 times a day every 6 hours.
  - 1. **Visit 2** (H+72 +/- 6 hours )

During the **Visit 2**, the following procedures will be performed:

- Record the vital status, the OMS scale, need for ventilator support…
- Record all concomitant medication use
- Record any AEs/SAEs, in particular infectious , hepatic and hematologic AE
- Record the length of time the patient has been in the prone position since Visit 1
- Collection of vital parameters: Temperature, Blood Pressure, pulse, respiratory rate, conscious, oxygen flow and saturation
- Perform a physical examination including the research of hepatomegaly and splenomegaly
- Perform blood test: blood count, platelets, C- Reactive Protein, creatinine + clearance (MDRD), ferritin, SGOT/SGPT, alkalines phosphatases, gammaGT, triglycerides, LDH, CPK, , fibrinogen, troponin, BNP, D-Dimers
- Perform serum and plasma bank sampling if possible in the center and after written authorization of the patient.

For the interventional group, the collection vital parameters, blood sampling and serum bank must be perform within 6 hours after the last infusion of charge dose.

For interventional group, the first infusion of maintenance dose will begin 12 hours after the last infusion of charge dose. During the period of maintenance, the infusion will be administered twice a day every 12 hours.

- - 1. **Visit 3** (Day 10 +/- 12 hours)

During the **Visit 3**, the following procedures will be performed:

- Record the vital status, the OMS scale, need for ventilator support…
- Record all concomitant medication use
- Record any AEs/SAEs, in particular infectious , hepatic and hematologic AE
- Record the length of time the patient has been in the prone position since Visit 2
- Collection of vital parameters: Temperature, Blood Pressure, pulse, respiratory rate, conscious, oxygen flow and saturation
- Perform a physical examination including the research of hepatomegaly and splenomegaly
- Perform blood test: blood count, platelets, C- Reactive Protein, creatinine + clearance (MDRD), ferritin, SGOT/SGPT, alkalines phosphatases, gammaGT, triglycerides, LDH, CPK, , fibrinogen, troponin, BNP, D-Dimers
- Perform serum and plasma bank sampling if possible in the center and after written authorization of the patient.

For the interventional group, the collection vital parameters, blood sampling and serum bank must be perform within 4 hours after the last perfusion of maintenance dose.

- - 1. **Visit 4** (Day 14 +/- 12hours)

During the **Visit 4**, the following procedures will be performed: **this visit will be performed** at hospital or by phone **if the patient is discharged from the hospital.**

- Record the vital status, the OMS scale, need for ventilator support…
- Record all concomitant medication use
- Record any AEs/SAEs, in particular infectious, hepatic and hematologic AE
- Record the length of time the patient has been in the prone position since Visit 3
- Collection of vital parameters: Temperature, Blood Pressure, pulse, respiratory rate, conscious, oxygen flow and saturation
- Perform a physical examination including the research of hepatomegaly and splenomegaly
- Perform blood test: blood count, platelets, C- Reactive Protein, creatinine + clearance (MDRD), ferritin, SGOT/SGPT, alkalines phosphatases, gammaGT, triglycerides, LDH, CPK, , fibrinogen, troponin, BNP, D-Dimers
- Perform serum and plasma bank sampling if possible in the center and after written authorization of the patient

**If the patient is discharged from hospital**, a blood test will be prescribed to perform, at home or at the laboratory, including blood count, platelets, C- Reactive Protein, creatinine + clearance (MDRD), ferritin, SGOT/SGPT, alkalines phosphatases, gammaGT, triglycerides, LDH, CPK, fibrinogen, troponin, BNP, and D-Dimers. The results will be recorder at the **Visit 4**. Phone visits must be organized to record condition status, concomitant medication and AEs/AES.

- - 1. **Visit 5** (Day 28 +/- 12 hours)

During the **Visit 5**, the following procedures will be performed: **this visit will be performed** at hospital or by phone **if the patient is discharged from the hospital.**

- Record the vital status, the OMS scale, need for ventilator support…
- Record all concomitant medication use
- Record any AEs/SAEs, in particular infectious, hepatic and hematologic AE
- Record the length of time the patient has been in the prone position since Visit 4
- Collection of vital parameters: Temperature, Blood Pressure, pulse, respiratory rate, conscious, oxygen flow and saturation
- Perform a physical examination including the research of hepatomegaly and splenomegaly
- Perform blood test: blood count, platelets, C- Reactive Protein, creatinine + clearance (MDRD), ferritin, SGOT/SGPT, alkalines phosphatases, gammaGT, triglycerides, LDH, CPK, fibrinogen, troponin, BNP, D-Dimers
- Perform serum and plasma bank sampling if possible in the center and after written authorization of the patient

**If the patient is discharged from hospital**, a blood test will be prescribed to perform, at home or at the laboratory, including blood count, platelets, C- Reactive Protein, creatinine + clearance (MDRD), ferritin, SGOT/SGPT, alkalines phosphatases, gammaGT, triglycerides, LDH, CPK,,fibrinogen, troponin, BNP, and D-Dimers. The results will be recorder at the **Visit 5**. Phone visits must be organized to record condition status, concomitant medication and AEs/AES.

**Flow Chart:**

**Serum and plasma banking**

**If the patient agree (specific consent form) blood samples** (1 dry and 1EDTA of 6 mL tubes, total 12mL) are collected on Day 0, day 3, Day 10 and Day 14 and 28 if the patient is hospitalized for the serum and plasma biobank (maximum 60mL for the 5 visits). They are sent to biological departments where they are centrifuged and aliquoted according to the laboratory manual. Serum and plasma will be stored in each center (at -80°C or -20°C if the previous option is not available) and they will be sent to the “Centre de Ressources Biologiques” CRB of CHRU Tours at the end of study.

This library will be used to evaluate the Anakinra efficacy and the disease severity by biomarkers assays (performed during a subsequent analysis, ancillary analysis).

The remaining samples after analyses may be used or transferred for other research work in order to improve scientific knowledge around the related problems to SARS-CoV-2 infection.

Research work using the samples may be carried out by the CHRU Tours team alone or in collaboration with other public or private partners, from national or international territory.

Analysis will include:

-Cytokine profile by multiplex (Meso Scale Discovery) including IL-1β, IL-6, IL1-7, IL-8, IL-10 and TNF α.

-Analysis of soluble urokinase-plasminogen activator receptor (suPAR) (Collaboration with **Dr** **Richard MACREZ** (Caen Universitary Hospital) and **Prof. Denis VIVIEN** (Unité INSERM UMR-U 1237, Physiopathology and Imaging of Neurological Disorders, Caen).

- 1. Patients care after the end of the participation

No exclusion period after the end of the trial participation. Patients will be excluded for participation in a therapeutic trial during their participation to the ANACONDA-COVID19 trial, but they will be authorized to participate in other observational studies or interventional no-therapeutic trials (e.g. French Covid-19).

- 1. Discontinuation and withdrawal

Once a participant is randomized into the study, every reasonable effort will be made in order to follow the participant throughout the entire study period even if there is a deviation from the intervention protocols, an early discontinuation of study treatment or if a participant misses one follow-up visit.

If a participant is lost-to-follow-up, every possible effort must be made by the study center personnel to contact the participant and determine the reason. The measures taken during follow-up must be documented.

A participant may discontinue study treatment at any time if the participant, the investigator, or the Sponsor feels that it is not in the participant’s best interest to continue. If a participant is withdrawn from treatment due to an adverse event or worsening, the participant will be followed and treated by the Investigator until the abnormal parameter or symptom has been resolved or stabilized. All participants who discontinue study treatment should be encouraged to complete all remaining scheduled visits and procedures. Early discontinuation of study treatment is not a reason for the participant to withdrawal from the study.

All participants are free to withdraw consent for participation at any time, for any reason, specified or unspecified, and without prejudice. Reasonable attempts will be made by the investigator to provide a reason for participant withdrawal. The reason for the participant’s withdrawal from the study will be specified in the participant’s source documents; in that event no further data will be collected for this participant. Nevertheless, data previously collected for this participant will be used if the patient has not expressly objected.

- 1. Discontinuation of the study

The study may be stopped definitely or temporarily at any time by the sponsor on the basis of information provided by the Data and Safety Monitoring Board (DSMB). **The DSMB will meet before the trial initiation, at one interim analysis (after the inclusion of 120 patients) for efficacy analysis and each 60 patients (60, 120, 180), for safety concerns.** The DSMB will also meet in case of a Suspected Unexpected Serious Adverse reaction (SUSAR), new facts (see Appendix 2), new safety information which could lead to a reevaluation of the benefit/risk ratio of the subject and/or new scientific information challenging study continuation. A safety report will be performed by vigilance unit at every 60 patients included and will be transmitted to the DSMB in case of new safety information.

**Anakinra Discontinuation :**

Patients must definitively discontinue study treatment if they experience any of the
following:

- Any medical condition that the investigator or Sponsor determines may jeopardize
  the patient's safety if he or she continues to receive study treatment
- Investigator or Sponsor determination that treatment discontinuation is in the best
  interest of the patient
- Pregnancy
- Severe allergic reaction to Anakinra

The primary reason for study treatment discontinuation should be documented on the
appropriate eCRF. Patients who discontinue study treatment will not be replaced.

The sponsor reserves the right to interrupt the study at any time if it appears that the inclusion objectives have not been met. In the event of early termination of the study, the information will be forwarded by the sponsor within 15 days to the French Health Authorities (ANSM) and to the (CPP) French committee of individual’s protection/ French protection of individuals committee.

- 1. Duration of the study

The recruitment will be completed within 4 months. Each participant will be followed up to day 28. Thus, the total duration of the study will be of maximum 4 months and 28 days (expected 3 months).

1. Randomization
   1. Sequence generation

Randomization will be performed at the end of the inclusion visit when all the eligibility criteria are checked.

Participants will be randomly assigned to receive either Anakinra plus optimized standard of care (oSOC) or oSOC alone in a 1:1 ratio as per a computer (SAS based) generated randomisation schedule. Randomization will be stratified on the **baseline CRP concentration** (<150 vs. >=150 mg/L), baseline **requirement of oxygen therapy** to maintain Sp02 >92% (3-6 liters per min vs. 7-10 liters per min) and **corticosteroid** therapy at baseline (< vs. >=0.5mg/kg/day prednisone) (yes vs no) using permuted blocks of random sizes. The block sizes will not be disclosed to study investigators.

- 1. Implementation

An allocation sequence will be generated by a statistician who is not involved in the recruitment or follow-up of the participants. In each centre, investigators will enrol and randomise patients.

- 1. Allocation concealment mechanism

Participants will be randomised using Ennov Clinical©, an online central randomisation procedure. To insure allocation concealment, the randomization procedure will not be possible until the participant has been recruited into the trial. Notably all selection criteria must be collected and met.

1. Blinding

Due to feasibility issues in the current emergency context, this trial is open label without placebo arm. As underlined by WHO, during the current pandemic, there are operational difficulties to obtain the preparation of placebos in a short time *(www.who.int/blueprint/15-01-2020-nfr-bp-wg-clinical-trials-ncov*). Thus, the patients, caregivers and outcome assessors will not be blinded from group allocation. Nevertheless, detection bias will be prevented by the use of an objective primary outcome.

1. Other strategies to reduce bias

In this trial, the primary outcome and most of the secondary outcomes are objective outcomes in order to avoid detection bias.

1. Data handling
   1. Data collection
      1. Access to data

The investigator will prepare and maintain adequate and accurate source documents designed to record all observations and other pertinent data for each participant of the study.

The sponsor is responsible for obtaining the agreement of all the parties involved in the study in order to guarantee direct access to source data, source documents and reports in all the sites where the study is being conducted, so that he can control their quality and audit them.

He is responsible for all information collected on participants enrolled in this study. All data collected during the course of this study must be reviewed and verified for completeness and accuracy by the Investigator.

- - 1. Source data and source document

Any original document or object helping to prove the existence or accuracy of a piece of information or fact recorded during the study is defined as a source document.

- 1. Data collection tool

Study personnel with their own access right to the study database, will enter/capture data from source documents corresponding to a participant into the protocol-specific electronic Case Report Form (eCRF).

All the information required by the protocol will be entered in the eCRF and an explanation will be provided for each missing piece of information. Data must be collected as they are obtained and transcribed into these forms in a clear manner.

If a correction is required for an eCRF, the time and date stamps track the person entering or updating eCRF data and create an electronic audit trail.

- 1. Confidentiality of data

In accordance with the regulatory provisions in force (Reglement EU 2016/679 RGPD and its transposition into French law LOI 2018-493, 20^th^ june 2018 from the Code de Santé Publique), people with direct access to source data will take all necessary precautions to ensure the confidentiality of information relating to study interventions, research studies and people taking part in them, particularly in regard to their identity and the results obtained. These people, such as investigators themselves, participate in professional secrecy.

During the study or once it is over, the information collected on the people taking part in it and forwarded to the sponsor by the investigators (or any other specialized staff member involved) will be made anonymous. Under no circumstances may the uncoded names or addresses of the people concerned appear in it.

For coding participants in the database or any study documents, the first letter of the first name and first letter of the last name of the participant will be recorded, accompanied by a code showing the order of inclusion of the participant in a centre.

The sponsor will ensure that each participant taking part in the study has given his agreement in writing for access to the individual data concerning him, which is strictly necessary for the quality control of the study.

- 1. Data management procedures

Data management will be performed by the INSERM CIC-P 1415. An eCRF will be developed using the Ennov Clinical® software. The management of eCRF will be done in agreement with the INSERM CIC-P 1415 Standardized Operating Procedures (SOP). The Clinical Research Associate in charge of the study will be trained to use the eCRF and will be in charge of the investigator’s training. Data will be entered in investigating centers through a secure web site, monitored by CRAs and potential queries will be edited by data managers, in agreement with a specified data management plan.

A data review will be done prior locking the database. The database will be locked in agreement with the INSERM CIC-P 1415 SOPs and data will be extracted in a SAS format or other, according to statistical requirements.

- 1. Data validation

Once data has been entered into the study database, a system of computerized data validation checks will be implemented and applied to the database on a regular basis. After an inconsistencies review, queries are entered, tracked, and resolved through the electronic data capture system directly (omissions and discrepancies will be forwarded to the investigator and CRA for resolution). The study database will be updated in accordance with the resolved queries. All changes will be documented.

- 1. Security and archival of data

The database is safeguarded against unauthorized access by established security procedures; appropriate backup copies of the database and related software files will be maintained.  Databases are backed up by the database administrator in conjunction with any updates or changes to the database.

1. Statistical considerations
   1. General principles of study analysis

Statistical analyses will be supervised by **Dr Agnès CAILLE** from the methodological and biostatistics unit, INSERM CIC-P 1415 University Hospital Center of Tours, 2 boulevard Tonnellé, 37044 TOURS Cedex.

A detailed analysis plan will be *a priori* defined.

SAS 9.4 and R 3.3.1 (or latest versions) software will be used.

A statistical report will be reported according to international guidelines: CONSORT (<http://www.consort-statement.org/> - Consultation: 2020.04.02).

A flow diagram will be done.

- 1. Analysis population definition

The ITT principle will be applied. Each patient will remain in the group assigned by randomisation, regardless of subsequent events.

The number of participants with missing data for each variable of interest will be indicated.

- 1. Baseline characteristics

Baseline characteristics will be reported per group using descriptive statistics. No statistical test will be performed on baseline measures.

- 1. Statistical analysis of the primary outcome

The number and proportion of patients with treatment success at Day 14, (defined as a patient alive and not requiring any of the following: Invasive mechanical ventilation (IMV) or Extracorporeal membrane oxygenation (ECMO)) will be reported in each group. We will estimate the between group risk difference, both point estimate and 95% confidence interval will be reported. The comparison of the proportion of treatment success between the two groups will be performed using a chi-square test.

The significance level associated with the interim and final analysis will be 0.0294.

Missing data for the primary outcome will be considered as a treatment failure, whatever the study group. Nevertheless, in this study, the likelihood that patients will be lost to follow-up is small.

We will also estimate the adjusted between group risk difference using a linear model (identity link function), adjustment variables will be stratification variables for randomization i.e. baseline CRP value (<150 vs. >=150 mg/l), baseline requirement of oxygen therapy to maintain Sp02 over 92% (3-6 liters per min vs. 7-10 liters per min) and corticosteroid therapy at baseline (> or =0.5mg/kg) (yes vs no).

- 1. Statistical analysis of secondary outcomes
- Treatment success (same definition as the Primary outcome) at Day 3, Day 7 and Day 28 will be analysed in the same manner as the primary outcome.
- OMS progression scale (on a 7-point ordinal scale): at Day 3, 10, 14 and 28. The number and proportion of patients in each category will be reported. Between-group comparison will be performed using a Cochran-Armitage test.
- Overall survival will be summarized using Kaplan-Meier curves and compared with the use of log-rank test
- Time to ICU admission and time to ventilatory support (ECMO, invasive mechanical ventilation, non-invasive ventilation, high flow oxygen therapy) will be analyzed using the competing risk approach, with death as competing risk
- Change from baseline to Day3, Day10, Day 14 and Day28 in NEWs score, HScore and inflammatory parameters will be described graphically using boxplots (one by study group at each visit) depicted on the same graph; changes over time will be compared between the two groups using mixed linear models, after data transformation if necessary
- Hospital length of stay: Time from inclusion to hospital discharge will be compared between the two groups using using the competing risk approach, with death as competing risk
- Number and proportion of patients with at least one serious adverse event during the study, particularly bacterial infection, septic shock, hepatic damage and neutropenia will be provided by study group and compared using a chi-square test

For those admitted in ICU only descriptive statistics will be provided:

- Number and proportion of patients with need for vasopressors will be reported.
- If IMV, Evolution of SpO2/FIO2 and PaO2/FiO2 ratio will be described graphically using boxplots (one by study group at each visit) depicted on the same graph;
- ICU length of stay: Time from admission in ICU to ICU discharge will be described.

No imputation of missing data will be performed on the secondary outcomes.

- 1. Additional analyses

Subgroup analyses

We plan to perform subgroup analyses on the stratification variables for randomization:

- Baseline CRP value (<150 vs. >=150 mg/l) (yes vs no).
- Baseline requirement of oxygen therapy to maintain Sp02 over 92% (3-6 liters per min vs. 7-10 liters per min)(yes vs no).
- Corticosteroid therapy at baseline (< vs. > =0.5mg/kg prednisone) (yes vs no).

Other sub group analysis will include :

- HScore baseline value <70 vs. >=70% (yes vs no).
- Ddimers count <2 000, >=2000 ng/ml (yes vs no).
- Lymphocytes count <500/mm3. >=500/mm3(yes vs no).

We will use of a linear model with an identity link function to estimate the risk differences in each subgroup as well as interaction p-values.

Identification of Predcitors of Anakinra efficacy

In cases where superiority of Anakinra will be shown the association between several clinical parameters at inclusion (including level of oxygen requirement, respiratory rate, temperature…), biological parameters (including, CRP, ferritin, LDH, lymphocyte count, eosinophil count, Ddimers, platelet count, polymorphonuclear count...), cytokines profile analysis with the primary end point will be explored using univariate and multivariate logistic regression models.

- 1. Interim analyses

We will perform one interim analysis, after inclusion of the first 120 patients, 60 in each study group. We will use Pocock’s boundary in order to stop prematurely the trial for efficacy:

- The p-value threshold associated with the interim analysis is 0.0294;
- The significance level associated with the final analysis is 0.0294.

This method yields an overall Type I error of 5%.

No stopping guidelines are defined to stop the trial prematurely for safety concerns but the DSMB will review safety data after the inclusion of 60, 120, and 180 patients.

- 1. Sample size

We plan to recruit **120 patients per group thus a total of 240 patients**. With alpha set to 5%, beta to 20%, a bilateral test, an estimated proportion of success in the control group of 80%, 216 patients are needed to show an absolute increase in the proportion of success of 13 percentage points in the experimental group (i.e. 93% of success). With one interim analysis, using Pocock’s approach, and applying a 1.11 inflation factor on the sample size, 240 patients are needed to achieve the 80% pre specified power and the p value boundary will be 0.0294 for both the interim and final analysis [19]. **The interim analysis will be performed on the first 120 randomized patients (60 per group)**. The hypothesis formulated for the proportion of success in the control group is based on epidemiological data obtained from Chinese cohort. Indeed, a large part of inpatients with COVID-19 experienced acute respiratory distress syndrome (ARDS) and around 20% of hospitalized patients need an admission in intensive care medicine to receive ventilation support [1-2].

1. Project feasibility

**47 COVID referent centers**, all over French territory will allow a national and rapid recruitment to conduct this trial. From 20 to 90 COVID-19 patients are actually hospitalized in each of the participating centers. The vast majority of French University Hospitals (CHU) including many « Assistance Publique - Hôpitaux de Paris » AP-HP centers and one military hospital « Hôpitaux d'Instruction des Armées » HIA agreed to participate to this trial. The « Hôpitaux Universitaires du Grand Ouest » HUGO neatwork has been strongly solicited.

The numbers of centers (47) will allow a rapid recruitment. Centers solicited routinely participate to clinical trials. Anakinra will be provided by SOBI. Swedish Orphan Biovitrum (SOBI) supply experimental drugs anakinra for the whole study – Written preliminary agreement has been obtained on the 27^th^ of March, 2020 by Dr Annik K-Laflamme, VP, Head of Medical Affairs Immunology and Specialty Care, SOBI (annik.laflamme@sobi.com).

End points will be updated according to the core outcome set, relying on the opinion of all users of research at the time of analysis of the study and will used validated scale/score for COVID-19 (but the primary outcome will not be modified). **Dr Sautenet**, MD-PhD in Tours University Hospital is part of the project and is one of the investigator of the COVID-19-COS international project which aims to to establish a core outcome set for trials in patients with confirmed or suspected COVID-19. The COVID-19-COS is aimed to be developed on April 2020 and will be implemented to this protocol in order to include relevant and consistent outcomes for this trial.

1. Expected results/benefits

So far, COVID-19, has infected more than 1.3 million peoples all around the world and caused the death of more than 74 000 patients. In France, as in others countries, number of patients infected and death is exponentially growing. To date, there is no efficient therapeutics to prevent or treat COVID-19. **Identifying a drug is a major concern and a public health emergency**. In addition, some French Hospitals, in particular in the eastern part of the country, are reaching saturation point and can no longer admit new patients in Intensive Care Unit. So that, identify an efficient drug to limit patient’s admission in ICU and to limit COVID-19 induced mortality is crucial. Favourable results could lead to new evidence-based recommendations in COVID-19.

1. Evaluation of security

Terminology used in this section is defined in Appendix 2.

- 1. Description of safety evaluation parameters

After analyzing the summary of product characteristics of Anakinra® and literature, the expected adverse reactions are particularly:

- Bacterial infections
- Hematologic effets with neutropenia and thrombopenia,
- Hepatic damages like increased liver enzymes (with cytolysis profile),
- Immunologic effects with anaphylaxis, and hypersensitivity reaction,
- Skin eruptions like rash and injection site reaction,
- Metabolic disorders like hypercholesterolemia,
- General disorders like arthralgia, headhache, diarrhea, nausea and vomiting

Throughout the study, the safety will be evaluated for each patient, in particular for the risks mentioned before.

The hepatic damages induced by Anakinra are usually self-limited, but cases with acute liver injury have been reported. The time of onset is within few weeks to up to 6 months after starting Anakinra. The typical clinical presentation resembled acute viral hepatitis, with a hepatocellular pattern of serum enzymes elevation. Most patients recovered within 2 to 8 weeks of stopping Anakinra. So, liver enzymes should be monitored as this treatment might cause acute cytolytic hepatitis.

Because the treatment period is short (10 days), we can exclude the risk of cancer (malignant melanoma and malignant lymphoma).

The data safety monitoring board (DSMB) will be systematically meet through teleconference at interim efficacy analysis (after the inclusion of 120 patients) and every 60 patients for safety (each 60, 120 and 180). It will also meet in case of Suspected Unexpected Serious Adverse reaction (SUSAR), new facts (see Appendix 2), new safety information which could lead to a reevaluation of the benefit/risk ratio of the subject and/or new scientific information challenging study continuation. A safety report will be performed by vigilance unit at every 60 patients included and will be transmitted to the DSMB in case of new safety information. The study may be stopped definitely or temporarily at any time by the sponsor on the basis of information provided by the DSMB

To ensure safety, the records of physical examination and the biological markers will be assessed (please see the details below).

- 1. Procedures and timing for the measurement, collection and analysis of the safety evaluation parameters
- Occurrence of serious adverse events during drug administration (Day 1 to day 10), hospital discharge, Day 14 and Day 28
- Liver and hematologic markers at Day 3, Day 10, Day 14 and Day 28
- Transminases
- Glutamate transferase
- Alkaline phosphatase
- Complete blood count in particular neutrophils count and platelets count
  1. Procedures in place for the documentation and the reporting of serious adverse events
     1. Investigator's responsibilities
        1. Reporting of serious adverse events
           1. Information to be reported to the sponsor

All serious adverse events occurring during the study will be reported as thorough as possible

by mail to the Vigilance Unit : uvrb@chu-tours.fr (Dr Céline LENGELLÉ, Marie-Sara AGIER or

Annie-Pierre JONVILLE-BERA : 02 47 47 80 37 or 02 47 47 85 92, 02 47 47 36 01), through the

serious adverse event reporting form (initial or follow-up declaration).

Transaminases>5N and neutrophils count < 1 G/L must be reported as serious adverse

event.

The following information must be transmitted:

- Participant identification (number, code, date ofbirth, date of inclusion, gender, weight, height)
- Seriousness criteria of the SAE
- Start and end date of the SAE,
- Clear and detailed description of the SAE (diagnosis, symptoms, intensity, timing, actions and results),
- Evolution of the SAE,
- Disease course or relevant participant history,
- Treatment received by the participant,
- Whether the adverse event is related to the medical product or to any associated treatments or other criteria.

Whenever possible, the investigator shall also attach report documents to the adverse event:

- A copy of the report of hospitalization or prolongation of hospitalization,
- A copy of the autopsy report (if applicable),
- A copy of all the results of additional tests, including those showing normal laboratory values,
- Any other documents, if necessary and appropriate.

These documents will be anonymized and coded with the identification number of the

participant.

- - - - 1. Procedure for SAE reporting to the sponsor

A report of every serious adverse event, regardless of whether the study procedures, investigational medicinal product or the research is suspected to have caused it (with the exception of those listed in the protocol as not requiring immediate reporting), will be reported by mail at the following address : uvrb@chu-tours.fr (Vigilance Unit).

A vigilance expert (Dr Céline LENGELLÉ, Marie-Sara AGIER or Annie-Pierre JONVILLE-BERA) can be reached by telephone (+ 33 2 47 47 80 37, + 33 2 47 47 85 92, + 33 2 47 47 36 01).

- - - - 1. Time limit for SAE reporting to the sponsor

The investigator has to report to the sponsor immediately (real time reporting) any serious adverse event that took place during the trial (with the exception of those listed in section 17.3.1.3 of the protocol which do not require immediate reporting).

This initial reporting must be provided in writing and should quickly be followed by a detailed written supplementary report.

- - - - 1. Reporting period of SAE to the sponsor

The investigator will record and report all serious adverse events that occur throughout the whole study, from the day that written informed consent is provided. This includes all events that occur during the follow-up period of the clinical trial, i.e. 28 days after the inclusion.

Moreover, all serious adverse events occurring after the study and that may be due to the research must be reported to the sponsor (e.g. serious events that may occur a long time after drug exposure, such as cancer or birth defects).

- - - 1. Reporting of non-serious adverse events

Non-serious adverse events, must be reported in the e-CRF with their date of occurrence, a description, their intensity evaluation (using the classification provided in Appendix 4) and duration, method of resolution, etiology, causal relationship (using the classification provided in Appendix 4) with the research and any decisions made.

- - - 1. Specificities of the protocol

Serious adverse events that do not need to be reported immediately include:

- Some circumstances requiring hospitalization that are not covered by the "hospitalization / prolongation of hospitalization" section under “serious adverse events" including:
- Hospitalization related to the study procedures and planned in the protocol,
- Admission for social or administrative reasons,
- Short hospital stays lasting less than 24 hours,
- Hospitalization for routine treatment or monitoring of the studied disease, which are not related to the deterioration of the participant's condition,
- Hospitalization for medical or surgical treatment scheduled before the start of the research.
  - 1. Sponsor's responsibilities
       1. Analysis of serious adverse events

The sponsor must evaluate:

- The causal relationship between serious adverse events according to ICH guidelines (as defined in Appendix 4) and the investigational medicinal product. If the investigator or the sponsor considers that a causal relationship may exist with the investigational medicinal product, then serious adverse events are considered to be suspected adverse reactions. If there is a difference in opinion between the sponsor and the investigator, both opinions are mentioned in the statement sent to the French Health Authorities (if a statement is required).
- The expected or unexpected features of the serious adverse reactions, using the reference document in force: the Summary of product Characteristics (SCP) on the first day of the period covered by the Development Safety Update Report (DSUR) or investigator’s brochure.

Adverse events for which the relationship with the medicinal product or study procedures is doubtful, possible, probable or highly probable will be considered to be related to the medicinal product or study procedures.

If they are unexpected, they will be classified as Suspected Unexpected serious Adverse Reactions (SUSAR) and notified in a report by the sponsor (see following paragraph).

- - - 1. Declaration of suspected unexpected serious adverse reactions

The sponsor will report all SUSAR to Eudravigilance (European pharmacovigilance database), the French Health Authorities (ANSM), and the investigators within the regulatory time limits for reporting:

- Immediately for fatal or life-threatening SUSAR. In such cases, additional relevant information should be sought and transmitted within a further period of 8 days.
- 15 calendar days maximum for all other serious unexpected effects. Additional relevant information should be sought and transmitted within a further period of 8 days.
  - - 1. Transmission of annual safety reports DSUR (Development Safety Update Report)

One year after the study is authorized by French Health Authorities, or at the request of French health Authorities, the sponsor will write a safety report containing:

- A safety analysis of participants included in the study,
- The list of serious adverse reactions (including expected and unexpected serious adverse reactions) that will have occurred in the trial both in France and abroad (including in non UE member countries) during the period covered by the report,
- The summary tables of all serious adverse events and serious adverse reactions that occurred in the trial since the beginning of the research.

This will be sent to French Health Authorities (ANSM) and to the ethics committee (CPP) within 60 days following the anniversary date of the study’s authorization.

- - - 1. Declaration of other safety data

The sponsor will notify the ANSM and the ethics committee (CPP) immediately of a new fact and if applicable the solutions taken.

Additional relevant information must be provided in new period of 8 days.

- - - 1. Data and Safety Monitoring Board (DSMB)

The DSMB is an independent advisory committee that discusses the benefit/risk ratio of the study and the implementation of a clinical trial with the sponsor and the coordinating investigator of the study.

It will consist of 4 members :

- - **Dr. Bénédicte LEBRUN-VIGNES** (Biological Pharmacology Department), La Pitié Salpetriere Hospital, APHP Paris, France
  - **Pr. Sophie GEORGIN-LAVIALLE** (Internal Medicine Department)Tenon hospital, APHP Paris, France
  - **Dr. Jean-Benoit HARDOUIN (**Department of Clinical Pharmacy - Public Health Epidemiology – Biostatistics), University hospital of Nantes, Nantes, France
  - **Pr. Renaud VERDON (**Department of Infectious Diseases), University hospital of Caen, Caen, France

It will systematically meet:

- For efficacy interim analysis (on the first 120 patients)
- For safety concerns (after each 60 inclusions)
- At any time if requested by the sponsor for each case of SUSAR
- Once a year, before sending DSUR to French Health Authorities
- If data may change the benefit and risk ratio during clinical trial.

At any time, the sponsor may refer to the DSMB to adjudicate whether an event is a SUSAR or a SAE when it is difficult to analyse or if new data changes the benefit and risk ratio during the clinical trial.

**A safety report will be performed by vigilance unit at every 60 patients included and will be transmitted to the DSMB in case of new safety information.**

- - 1. In utero exposure

All women at age of motherhood will be systemically tested with a pregnancy detection test at time of enrolment. In case of the test is positive, the woman will be excluded of the trial.

If a woman becomes pregnant during the clinical trial, the pregnancy should be reported to the sponsor within the three months of follow up.

The investigator will then complete the serious adverse event reporting form to inform the sponsor's vigilance unit. This form must contain the expected date of delivery, the contact details of the obstetrician and the expected maternity hospital of delivery if the pregnancy continues.

The investigator has to follow the participant until the end of the pregnancy or until its interruption and notify the outcome to the sponsor through a standardized form completed after the pregnancy.

If the outcome of pregnancy involves a serious adverse event (spontaneous abortion before hospitalization, foetal death, birth defect...), the investigator has to follow the procedure for the reporting of serious adverse effects.

In cases of paternal exposure, the investigator must obtain consent from the expectant mother before collecting information about the pregnancy.

1. Practical issues on study sites

A clinical research technician will be responsible for:

- Logistics of the study,
- Producing reports concerning its state of progress,
- Ensuring e-CRF completion and update (request for additional information, corrections, etc.)
- Sending biological samples,
- Transmitting SAEs to the sponsor.

She will work in accordance with the SOP, in cooperation with the clinical research associate appointed by the sponsor.

1. Quality control – Monitoring visits

A clinical research associate appointed by the sponsor will regularly visit each study centre according to the monitoring plan depending on the frequency of inclusions and at the end of the study. During these visits, the following aspects will be reviewed:

- Written informed consent,
- Compliance with the study protocol and the procedures set out in it,
- Quality of the data collected in the case report form: its accuracy, missing data, consistency of the data with the source documents (medical records, appointment diaries, the originals of laboratory results etc.),
- Adequate management of medicinal products.

Each monitoring visit will be performed according to the risks assessment and the monitoring plan and then, a monitoring report will be written.

1. Audit and inspection

An audit may be performed at any time by people appointed by the [sponsor who are](http://www.chusa.jussieu.fr/urcest/sous_cadre.php?fich=Lexique/new_index.php?isphp=0&fich=EC/legislation/DispositionslegislativesPromoteur.htm) independent to those responsible for the study. The audit’s aim is to ensure the good quality of the study and that the law and regulations in force are being accounted for.

The investigators agree to comply with the requirements of the sponsor and the relevant authority for an audit or an inspection of the study.

The audit can apply to all stages of the study, from development of the protocol to publication of the results, filing the data used or produced in the study.

1. Storage of documents and data at the end of the study

The following documents relating to the study are archived in accordance with Good Clinical Practice:

- 1. By the investigators:
- In accordance to the regulatory provisions in force:
- The protocol and any amendments to the protocol.
- The case record forms.
- The source files of participants who signed a consent form.
- All other documents and letters relating to the study.
- The original copies of informed consent forms signed by participants

The investigator is responsible for all these documents during the regulation period of archiving.

- 1. By the sponsor:
- In accordance to the regulatory provisions in force:
- The protocol and any amendments to the protocol.
- The originals of the case record files.
- All other documents and letters relating to the study.
- A copy of the informed consent forms signed by the participants
- Documents relating to serious adverse events

The sponsor is responsible for all these documents during the regulation period of archiving.

No removal or destruction may be carried out without the sponsor's agreement. At the end of the regulatory archiving period, the sponsor will be consulted regarding destruction. All the data, documents and reports could undergo audit or inspection.

1. Administrative, ethical and regulatory considerations

The sponsor and the investigator or investigators undertake to conduct ~~this~~ the study in compliance with the French law in force (Code de Santé Publique), the recommendations of French and international Good Clinical Practices (ICH), the Helsinki Declaration (Ethical Principles for Medical Research involving Human Subjects), and the European regulations related to clinical research.

The study will be conducted in accordance with this protocol. With the exclusion of emergency situations necessitating specific therapeutic actions to take place, the investigators guarantee to follow the protocol in all respects, in particular in regards to obtaining consent and the reporting and follow-up of serious adverse events.

This research is registered in the European EudraCT database under n° 2020-001734-36 in accordance with art. L1121.15 of the French Public Health Act.

- 1. Information and consent forms

Participants will be informed of the objectives of the study and their informed sign consent will be obtained. Participants care will not be affected by their decision to participate or not in the study.

- 1. CNIL

The data recorded in this study will undergo computer processing by INSERM CIC-P 1415 – University Hospital Center of Tours, and these data collected during the trial will be processed in accordance with the requirements of the CNIL and General Data Protection Regulation (GDPR)).

- 1. Research ethics committee

The protocol, informed consent form and participant information sheet will be reviewed and approved by a French ethics committee (CPP) prior to study initiation.

- 1. Regulatory authorities

The sponsor undertakes that this protocol will submit an application for a clinical trial authorization from the ANSM prior to study initiation.

The Coordinating Investigator will regularly provide to the Ethics Committee and Regulatory Authorities (ANSM), any reports, updates or appropriate information (e.g.., amendments, administrative letters, Adverse Events reports) according to regulatory requirements. Deviations or protocol amendments should not be initiated without a prior written approval from the Ethics Committee and from the Regulatory Authorities.

- 1. Biological collection

The collection of physiological samples to be undertaken for this study will be declared to the Ethics Committee at the same time as the request is made to authorise the study. After the study, conservation of the collection of physiological samples will be declared to the Minister of Research and to the director of the Regional Hospitalisation Agency.

- 1. Protocol amendments

A substantial modification is a modification of a nature that is likely to have a significant impact on: the safety of the people involved, the conditions of validity and the results of the study, the quality and safety of the investigational medicinal products as well as on the interpretation of the scientific documents. A written amendment must be submitted to the sponsor prior to its implementation, the latter must obtain approval from the ethics committee and authorisation from ANSM.

Non-substantial modifications, i.e. those not having a significant impact on any aspect of the study whatsoever, will be communicated to the ethics committee for information purposes.

Any amendments to the protocol must be made known to all the investigators participating in the study. The investigators undertake to comply with the contents.

Any amendment modifying the management of participants or the benefits, risks or constraints of the study will require a new Participant Information and Informed Consent form, which must be completed and collected according to the same procedure as used for the previous one.

- 1. Registration

The study protocol will be registered on ClinicalTrials.gov before recruitment of the first trial participant. Recorded data will be updated regularly. At the end of the trial, the results of the trial will also be posted in the Results section of the registry.

- 1. Insurance

The University Hospital Center of Tours is the sponsor of this study and will take out an insurance policy covering third party liability with SHAM complying with the provisions of article L1121-10 of the French Public Health Act.

1. Dissemination policy
   1. Authorship

Any written or oral communication of the results of the study will be previously agreed by the coordinating investigator and, if necessary, by the scientific committee constituted for the study.

Publication of the main results will mention the sponsor and the funding source. We will follow the Recommendations for the Conduct, Reporting, Editing, and Publication of Scholarly Work in Medical Journals (updated in December 2014) from the International Committee of Medical Journal Editors (ICMJE). All investigators not-cited in the authorship will be listed as non-author contributors.

- 1. Communication of the results to participants

In accordance with the regulatory provisions in force, participants will be informed, at their request, of the overall results of the study.

- 1. Study data sharing statement

Open science aims to share scientific information almost in real-time online and free of charge to the user by providing free access to research publications and open access to scientific data. Especially in the current emergency context, this concept is of primary importance to increase the collective response of researchers. We will propose a data sharing plan within the respect of the European regulation (RGPD).

1. Financial data
   1. Budget of the study

The study budget will be coordinated by the sponsor, the university hospital center of Tours. A financial convention will be signed between each participating center and the sponsor.

- 1. Compensation for participants

There will be no compensation for trial participants.

REFERENCES

[1] Huang C, Wang Y, Li X, Ren L, Zhao J, Hu Y, et al. Clinical features of patients infected with 2019 novel coronavirus in Wuhan, China. Lancet 2020;395:497–506.

[2] Wang D, Hu B, Hu C, Zhu F, Liu X, Zhang J, et al. Clinical Characteristics of 138 Hospitalized Patients With 2019 Novel Coronavirus-Infected Pneumonia in Wuhan, China. JAMA 2020.

[3] [Chen N](https://www-ncbi-nlm-nih-gov.proxy.insermbiblio.inist.fr/pubmed/?term=Chen N%5BAuthor%5D&cauthor=true&cauthor_uid=32007143), [Zhou M](https://www-ncbi-nlm-nih-gov.proxy.insermbiblio.inist.fr/pubmed/?term=Zhou M%5BAuthor%5D&cauthor=true&cauthor_uid=32007143), [Dong X](https://www-ncbi-nlm-nih-gov.proxy.insermbiblio.inist.fr/pubmed/?term=Dong X%5BAuthor%5D&cauthor=true&cauthor_uid=32007143) et all. Epidemiological and clinical characteristics of 99 cases of 2019 novel coronavirus pneumonia in Wuhan, China: a descriptive study [Lancet.](https://www-ncbi-nlm-nih-gov.proxy.insermbiblio.inist.fr/pubmed/?term=Chen+N%2C+Zhou+M%2C+Dong+X%2C+Qu+J%2C+Gong+F%2C+Han+Y%2C+et+al.+Epidemiological+and+clinical+characteristics+of+99+cases+of+2019+novel+coronavirus+pneumonia+in+Wuhan) 2020 Feb 15;395(10223):507-513.

[4] Ruan Q, Yang K, Wang W, Jiang L, Song J. Clinical predictors of mortality due to COVID-19 based on an analysis of data of 150 patients from Wuhan, China. Intensive Care Med 2020; published online March 3.

[5] [Shakoory B](https://www-ncbi-nlm-nih-gov.proxy.insermbiblio.inist.fr/pubmed/?term=Shakoory B%5BAuthor%5D&cauthor=true&cauthor_uid=26584195), [Carcillo JA](https://www-ncbi-nlm-nih-gov.proxy.insermbiblio.inist.fr/pubmed/?term=Carcillo JA%5BAuthor%5D&cauthor=true&cauthor_uid=26584195), [Chatham WW](https://www-ncbi-nlm-nih-gov.proxy.insermbiblio.inist.fr/pubmed/?term=Chatham WW%5BAuthor%5D&cauthor=true&cauthor_uid=26584195) et al.Interleukin-1 Receptor Blockade Is Associated With Reduced Mortality in Sepsis Patients With Features of Macrophage Activation Syndrome: Reanalysis of a Prior Phase III Trial. [Crit Care Med.](https://www-ncbi-nlm-nih-gov.proxy.insermbiblio.inist.fr/pubmed/?term=Interleukin-1+receptor+blockade+is+associated+with+reduced+mortality+in+sepsis+patients+with+features+of+the+macrophage+activation+syndrome%3A) 2016 Feb;44(2):275-81.

[6] Serbina, N. V., Jia, T., Hohl, T. M. & Pamer, E. G. Monocyte-mediated defense against microbial pathogens. Annu. Rev. Immunol. 26, 421–452 (2008).

[7]https://www.biorxiv.org/content/10.1101/2020.02.12.945576v1.full.pdf

[8]https://www.medrxiv.org/content/10.1101/2020.03.24.20042655v1.full.pdf

[9]<https://www.medrxiv.org/content/10.1101/2020.02.23.20026690v1.full.pdf>

[10] Garlanda, C., Dinarello, C. A. & Mantovani, A. The interleukin-1 family: back to the future. Immunity 39, 1003–1018 (2013).

[11] [Pocock SJ](https://en.wikipedia.org/wiki/Stuart_Pocock) (1977). "Group sequential methods in the design and analysis of clinical trials". Biometrika. 64 (2): 191–9.

[12] [Spångfors M](https://www.ncbi.nlm.nih.gov/pubmed/?term=Spångfors M%5BAuthor%5D&cauthor=true&cauthor_uid=27386753), Arvidsson L, [Karlsson V](https://www.ncbi.nlm.nih.gov/pubmed/?term=Karlsson V%5BAuthor%5D&cauthor=true&cauthor_uid=27386753), [Samuelson K](https://www.ncbi.nlm.nih.gov/pubmed/?term=Samuelson K%5BAuthor%5D&cauthor=true&cauthor_uid=27386753).The National Early Warning Score: Translation, testing and prediction in a Swedish setting. [Intensive Crit Care Nurs.](https://www.ncbi.nlm.nih.gov/pubmed/27386753) 2016 Dec;37:62-67.

[13] [Fardet L](https://www.ncbi.nlm.nih.gov/pubmed/?term=Fardet L%5BAuthor%5D&cauthor=true&cauthor_uid=24782338), [Galicier L](https://www.ncbi.nlm.nih.gov/pubmed/?term=Galicier L%5BAuthor%5D&cauthor=true&cauthor_uid=24782338), [Lambotte O](https://www.ncbi.nlm.nih.gov/pubmed/?term=Lambotte O%5BAuthor%5D&cauthor=true&cauthor_uid=24782338), [Marzac C](https://www.ncbi.nlm.nih.gov/pubmed/?term=Marzac C%5BAuthor%5D&cauthor=true&cauthor_uid=24782338), [Aumont C](https://www.ncbi.nlm.nih.gov/pubmed/?term=Aumont C%5BAuthor%5D&cauthor=true&cauthor_uid=24782338), [Chahwan D](https://www.ncbi.nlm.nih.gov/pubmed/?term=Chahwan D%5BAuthor%5D&cauthor=true&cauthor_uid=24782338), [Coppo P](https://www.ncbi.nlm.nih.gov/pubmed/?term=Coppo P%5BAuthor%5D&cauthor=true&cauthor_uid=24782338), [Hejblum G](https://www.ncbi.nlm.nih.gov/pubmed/?term=Hejblum G%5BAuthor%5D&cauthor=true&cauthor_uid=24782338). Development and validation of the HScore, a score for the diagnosis of reactive hemophagocytic syndrome. [Arthritis Rheumatol.](https://www.ncbi.nlm.nih.gov/pubmed/?term=HScore+and+lambotte) 2014 Sep;66(9):2613-20.

[14] Granowitz E et al. Pharmacokinetics, safety, and immunomodulatory effects of human recombinant interleukin-1 receptor antagonist in healthy humans. CYTOKINE 1992;4(5):353-360.

[15] Badheka A et al. Use of an interleukin-1 receptor antagonist for suspected sepsis with hyperinflammation in children. Crit Care Med. 2019; 48:1561.

[16] Opal S et al. Confirmatory interleukin-1 receptor antagonist trial in severe sepsis: A phase III, randomized, double-blind, placebo-controlled, multicenter study. Crit Care Med. 1997; 25:1115-1124.

[17] Opal S et al. Confirmatory interleukin-1 receptor antagonist trial in severe sepsis: A phase III, randomized, double-blind, placebo-controlled, multicenter study. Crit Care Med. 1997; 25:1115-1124.

[18] Fisher C et al. Initial evalutation of human recombinant interleukin-1 receptor antagonist in the treatment of sepsis syndrome: A randomized, open-label, placebo-controlled multicenter study. Crit Care Med 1994; 22(1):12-21.

[19] Jennison, Christopher. Group Sequential Methods with Applications to Clinical Trials. Boca Raton: Chapman & Hall/CRC, 2000.

APPENDIX

1. List of study sites

**ASSOCIATED INVESTIGATORS/RESEARCHERS n=47**

| **Institution name** | **Family name** | **Given name** | **Adress** | **Mail** |
| --- | --- | --- | --- | --- |
| APHP BROCA | HANON | Olivier | Hôpital Broca 54-56 rue Pascal  75013 Paris | olivier.hanon@aphp.fr |
| APHP LA PITIE SALPETRIERE | CACOUB | Patrice | Hôpital de la Pitié Salpétriere 83 bd de l'hôpital 75651 Paris Cedex 13 | [patrice.cacoub@aphp.fr](mailto:patrice.cacoub@aphp.fr) |
| APHP ST ANTOINE | MEKINIAN | Arsène | Hôpital St Antoine 184 rue du Faubourg Saint Antoine 75012 Paris | [arsene.mekinian@aphp.fr](mailto:arsene.mekinian@aphp.fr) |
| AHPH JEAN VERDIER | BOURGARIT-DURAND | Anne | Hôpital Jean Verdier Avenue du 14 juillet 93143 Bondy Cedex | [anne.bourgaritdurand@aphp.fr](mailto:anne.bourgaritdurand@aphp.fr) |
| CHU ANGERS | URBANSKI | Geoffroy | CHU Angers 4 rue Larrey  49100 Angers | [geoffrey.urbanski@chu-angers.fr](mailto:geoffrey.urbanski@chu-angers.fr) |
| CHU BORDEAUX | RIVIERE | Etienne | CHU Bordeaux Groupe Hospitalier Sud - Hôpital Haut Lévêque Avenue de Magellan 33604 Pessac | [riviere.etienne@chu-bordeaux.fr](mailto:riviere.etienne@chu-bordeaux.fr) |
| CHU GRENOBLE | BOUILLET | Laurence | CHU Grenoble  Avenue Maquis du Grésivaudan 38700 La Tronche | [Lbouillet@chu-grenoble.fr](mailto:Lbouillet@chu-grenoble.fr) |
| CHU LIMOGES | FAUCHAIS | Anne-Laure | CHU Limoges 2 Avenue Martin Luther King 87000 Limoges | [anne-laure.fauchais@unilim.fr](mailto:anne-laure.fauchais@unilim.fr) |
| CHU NANTES | RAFFI | François | CHU Nantes Hôpital Hôtel Dieu Place Alexis Ricordeau 44093 Nantes Cedex 1 | [francois.raffi@chu-nantes.fr](mailto:francois.raffi@chu-nantes.fr) |
| CHU NICE | COURJON | Johan | Hôpital Archet 1 CS 23079 151 route de St Antoine de Ginestière 06202 NICE cedex 3 | [courjon.j@chu-nice.fr](mailto:courjon.j@chu-nice.fr) |
| CHU REIMS | SERVETTAZ | Amélie | CHU Reims Rue Cognacq Jay  51092 Reims | [amelie_servettaz@yahoo.fr](mailto:amelie_servettaz@yahoo.fr) |
| CHU TOURS | AUDEMARD-VERGER | Alexandra | CHU Tours Bd Tonnellé 37044 Tours Cedex 2 | [a.audemardverger@chu-tours.fr](mailto:a.audemardverger@chu-tours.fr) |
| CH AIX EN PROVENCE | MAULIN | Laurence | CH Aix en Provence Avenue des Tamaris  13100 Aix-en-Provence | [lmaulin@ch-aix.fr](mailto:lmaulin@ch-aix.fr) |
| CH BLOIS | LIOGER | Bertrand | Centre Hospitalier Simone Veil de Blois  Mail Pierre Charlot  41016 Blois | [liogerb@ch-blois.fr](mailto:liogerb@ch-blois.fr) |
| CH CORNOUAILLE | TALARMIN | Jean-Philippe | CHI de Cornouaille  14 bis avenue Yves Thépot  29107 QUIMPER cedex | [jp.talarmin@ch-cornouaille.fr](mailto:jp.talarmin@ch-cornouaille.fr) |
| CH INTERCOMMUNAL POISSY | DEVAUX | Mathilde | 10 rue du Champ Gaillard 78100 Saint-Germain-en-Laye | [mathilde.lm.devaux@gmail.com](mailto:mathilde.lm.devaux@gmail.com) |
| CH LA ROCHE SUR YON | GUIMARD | Thomas | Centre Hospitalier Départemental Vendée Boulevard Stéphane Moreau 85000 La Roche-sur-Yon | [thomas.guimard@chd-vendee.fr](mailto:thomas.guimard@chd-vendee.fr) |
| CHR ORLEANS | PRAZUK | Thierry | CHR Orléans  14 Avenue de l'Hôpital 45100 Orléans | [thierry.prazuck@chr-orleans.fr](mailto:thierry.prazuck@chr-orleans.fr) |
| CH VALENCIENNES | WAYENBERG | Laura | CH Valenciennes 114 Avenue Desandrouin 59300 Valenciennes | [wayenberg-l@ch-valenciennes.fr](mailto:wayenberg-l@ch-valenciennes.fr) |
| HÔPITAL DE LA CROIX ROUSSE | JAMILLOUX | Yvan | Hôpital de la Croix Rousse103 grande rue de la Croix Rousse69004 LYON | [yvan.jamilloux@chu-lyon.fr](mailto:yvan.jamilloux@chu-lyon.fr) |
| HÔPITAL D'INSTRUCTION DES ARMEES DE PERCY | ALETTI | Marc | HIA Percy 2 Rue du Lieutenant Raoul Batany 92140 Clamart | [marc.aletti@free.fr](mailto:marc.aletti@free.fr) |
| HÔPITAL FOCH | GROH | Matthieu | Hôpital Foch 40 Rue Worth 92150 Suresnes | [m.groh@hopital-foch.com](mailto:m.groh@hopital-foch.com) |
| HÔPITAL EUROPEEN DE MARSEILLE | CHICHE | Laurent | Hôpital Européen de Marseille 6 Rue Désirée Clary 13003 Marseille | l.chiche@hopital-europeen.fr |
| HÔPITAUX PRIVES DE METZ | MAURIER | François | Hôpital Belle Ile de Metz 2 Rue Belle-Isle 57000 Metz | [francois.maurier@hp-metz.fr](mailto:francois.maurier@hp-metz.fr) |
| GROUPEMENT HOSPITALIER ST JOSEPH | EMMERICH | Joseph | GH St Jospeh 185 Rue Raymond Losserand 75014 Paris | [jemmerich@hpsj.fr](mailto:jemmerich@hpsj.fr) |
| PÔLE SANTE LEONARD DE VINCI | AUDEMARD-VERGER | Hélène | PSLV 1 Avenue Alexandre Minkowski 37170 Chambray-lès-Tours | helene.verger7@hotmail.fr |
| CHU BREST | LEMOIGNE | Emmanuelle | BREST Hôpital de la Cavale Blanche Bd T Prigent  29609 Brest Cedex | [emmanuelle.lemoigne@chu-brest.fr](mailto:emmanuelle.lemoigne@chu-brest.fr) |
| CHU GRENOBLE | EPAULARD | Olivier | CHU Grenoble Pôle Médecine Aiguë et Urgences (PUMA) BP 217 38043 Grenoble Cedex 09 | [oepaulard@chu-grenoble.fr](mailto:oepaulard@chu-grenoble.fr) |
| CHU NICE | QUEYREL-MORANNE | Viviane | Hôpital Pasteur 2 30 rue voie romaine06200 Nice | [queyrel-moranne.v@chu-nice.fr](mailto:queyrel-moranne.v@chu-nice.fr) |
| APHP LA PITIE SALPETRIERE | COHEN | Fleur | Hôpital de la Pitié Salpétriere 83 bd de l'hôpital 75651 Paris Cedex 13 | [fleur.cohen@aphp.fr](mailto:fleur.cohen@aphp.fr) |
| CLINIQUE TESSIER | LEROY | Vincent | Clinique Tessier  118 Avenue Desandrouin 59300 Valenciennes | [vileroy@ahnac.com](mailto:vileroy@ahnac.com) |
| CHU TOURS | MARCHAND-ADAM | Sylvain | CHU de Tours 2 bd Tonnelé  37044 Tours Cedex 2 | s.marchandadam@univ-tours.fr |
| CHU TOURS | BERNARD | Louis | CHU de Tours 2 bd Tonnelé  37044 Tours Cedex 2 | l.bernard@chu-tours.fr |
| APHM - CHU TIMONE | BRIANTAIS | Antoine | Hôpital de la Timone 278 rue Saint Pierre 13005 Marseille | [Antoine.BRIANTAIS@ap-hm.fr](mailto:Antoine.BRIANTAIS@ap-hm.fr) |
| CHU NICE | LEROY | Sylvie | CHU Nice  30 voie romaine 06002 NICE | [leroy.s2@chu-nice.fr](mailto:leroy.s2@chu-nice.fr) |
| CH AVIGNON | PESTRE | Vincent | CH Avignon 305 Rue Raoul Follereau 84000 Avignon | [pestre.vincent@ch-avignon.fr](mailto:pestre.vincent@ch-avignon.fr) |
| CHU LIMOGES | FAUCHER | Jean-François | CHU Limoges 2 Avenue Martin Luther King 87000 Limoges | [jean-francois.faucher@unilim.fr](mailto:jean-francois.faucher@unilim.fr) |
| CHU LIMOGES | FOURCADE | Laurent | CHU Limoges 2 Avenue Martin Luther King 87000 Limoges | [laurent.fourcade@unilim.fr](mailto:laurent.fourcade@unilim.fr) |
| CH SAINT QUENTIN | DOUADI | Yousef | CH St Quentin1 Avenue Michel de l'Hospital BP 60802321 Saint-Quentin | [ydouadi@free.fr](mailto:ydouadi@free.fr) |
| CHU CAEN | AOUBA | Achille | CHU Caen  Avenue de la Côte de Nacre 14000 Caen | [aouba-a@chu-caen.fr](mailto:aouba-a@chu-caen.fr) |
| HÔPITAL SAINT JOSEPH | BIENVENU | Boris | Hôpital Saint Joseph 26, boulevard de Louvain 13285 Marseille Cedex 8 | [bbienvenu@hopital-saint-joseph.fr](mailto:bbienvenu@hopital-saint-joseph.fr) |
| CH LE HAVRE | LANGLOIS | Vincent | CH le Havre 29 Avenue Pierre Mendès 76290 Montivilliers | [vincent.langlois@ch-havre.fr](mailto:vincent.langlois@ch-havre.fr) |
| CH LISIEUX | GEFFRAY | Loic | Centre Hospitalier Robert Bisson 4 Rue Roger Aini 14107 Lisieux | [l.geffray@ch-lisieux.fr](mailto:l.geffray@ch-lisieux.fr) |
| CHU TOURS | FOUGERE | Bertrand | CHU Tours 101 Bd Henri Barbusse 37044 Tours Cedex | damien.ricard@m4x.org |
| CHU ANGERS | ANNWEILER | Cédric | CHU Angers 4 rue Larrey  49100 Angers | [ceannweiler@chu-angers.fr](mailto:ceannweiler@chu-angers.fr) |
| CHU ANGERS | MAHIEU | Rafael | CHU Angers 4 rue Larrey  49100 Angers | [rafael.mahieu@chu-angers.fr](mailto:rafael.mahieu@chu-angers.fr) |
| CHU NIMES | GOULABCHAND | Radjiv | CHU Nimes Hôpital Carémeau Place du Pr Debré 30029 Nîmes Cedex 9 | [Radjiv.GOULABCHAND@chu-nimes.fr](mailto:Radjiv.GOULABCHAND@chu-nimes.fr) |

1. Safety evaluation terminology

- **Adverse Event (AE)** (article R.1123-46of the French Public Health Act)**:** any harmful event occurring in a person taking part in a research study involving individuals, whether or not that event is linked to the study or to the product being investigated in the study.
- **Serious Adverse Event (SAE)** (article R.1123-46 of the Public Health Act and the ICH E2B guide): the severity is defined by one of the following observations:
- death,
- life-threatening for the person taking part in the research study (directly life-threatening at the time of the event, regardless of the consequences of corrective or palliative therapy),
- disability or significant or lasting handicap,
- hospitalization,
- prolongation of hospitalization,
- malformation/birth defect,
- potentially serious event (adverse clinical event or laboratory test result considered serious by the investigator).
- **Adverse Reaction (AR)**: any untoward and unintended reaction to an investigational medicinal product, whatever the dose administered.
- **Serious Adverse Reaction (SAR)**: serious adverse events potentially caused by a medicinal product.
- **Suspected Unexpected Serious Adverse Reaction** (**SUSAR**) (article R.1123-46 of the French Public Health Act): serious adverse reaction, the type, severity, frequency or outcome in which it is inconsistent with the information contained in the summary of product characteristics for an authorized medicinal product or, in the case of an unauthorized medicinal product, in the investigator’s brochure.
- **New fact**(article R.1123-46 12°of the French Public Health Act) **:** New information which could lead to:
  - A re-evaluation of the benefit/risk ratio of the study or the investigational product,
  - Modifications, due to its foreseen sufficiency in making changes in the way the product is used, to documents concerning the study, or if necessary to the way the study is conducted,
  - A suspension, interruption or modification of the research protocol or similar research.
- **Causal relationship:** relationship between the adverse event and the treatment. An adverse event related to an investigational medicinal product will be classified as an adverse reaction. Factors to consider when determining the cause of an adverse event are:
- the chronological order of events,
- the disappearance of the AE at the time of drug discontinuation and/or the reappearance upon re-administration,
- the pharmacodynamic and pharmacokinetic properties of the drug,
- history of a similar event occurring during the administration of the drug or a drug of the same class,
- other potential causes of the AE.

1. Severity evaluation of non-serious adverse events

| **Severity (Toxicity Grade)** | **Description** |
| --- | --- |
| Mild | Transient or mild discomfort; no limitation in activity; no medical intervention or therapy required. The participant may be aware of the sign or symptom but tolerates it reasonably well. |
| Moderate | Mild to moderate limitation in activity, no or minimal medical intervention/therapy required. |
| Severe or life threatening | Marked limitation in activity, medical intervention/therapy required, hospitalizations possible.  The participant is at risk of death due to the adverse experience as it occurred. This does not refer to an experience that hypothetically might have caused death if it were more severe. |

1. Causal relationship evaluation

In accordance with ICH guidelines on the management of adverse events in clinical trials- ICHE2B(R3)12 May 2005 version- the relationship between all notified SAE and the research must be assessed.

The method used to evaluate the relationship of the event is as follows:

- **Unrelated**: the event occurred within a time period that is not compatible with the administration of the medicinal product, and/or sufficient information exists showing that the observed reaction is unrelated to the medicinal product, and/or a probable alternative explanation exists.
- **Doubtful**: the timing of the event (occurrence, outcome) is inconsistent with the administration of the medicinal product. The event is most likely related to factors other than the medicinal product such as the participant’s clinical condition or concomitant administration of other medicinal products.
- **Possible**: the event occurred within a period that is compatible with the administration of the medicinal product. Although a causal effect of the product cannot be ruled out, other factors can be implicated, such as the participant’s clinical condition or the concomitant administration of other medicinal products. Information about the outcome upon discontinuation of the studied treatment can be absent or inconclusive.
- **Probable**: the event occurred within a period that is compatible with the administration of the medicinal product. It cannot reasonably have been caused by another factor, such as the participant’s clinical condition or the concomitant administration of other medicinal products. The outcome upon discontinuation of the medicinal product must be clinically compatible. Information about re-challenge with the medicinal product is not essential.
- **Highly probable**: the event occurred within period that is highly compatible with the administration of the medicinal product. It cannot be explained by another factor such as the participant’s clinical condition or the concomitant administration of other medicinal products. The outcome upon discontinuation of the medicinal product must be clinically compatible. The event should have a pharmacological or pathophysiological explanation, or recurs upon re-challenge with the medicinal product.
