# Supplementary material for: Efficacy and safety of anakinra in adults presenting deteriorating respiratory symptoms from COVID-19: A randomized controlled trial
Source: PLoS One. 2022 Aug 4;17(8):e0269065. doi: 10.1371/journal.pone.0269065 (PMC9351999; doi:10.1371/journal.pone.0269065)
Supplement: S2 File — (DOCX) [file pone.0269065.s002.docx]

|  | **Statistical analysis plan** | **CIC de Tours** |
| --- | --- | --- |

**Efficacy and safety of ANAkinra during**

**Adult « COVID-19 » with Aggravating**

**respiratory symptoms: a multicenter open-label controlled randomized trial**

**ANACONDA**

***Final statistical analysis plan***

Version 1 – 15.12.2020

**Dr Audemard - Verger**

Department of Internal Medicine and Clinical Immunolgy

University Hospital Center of Tours

**Document written by:** Amélie Le Gouge

**Document revised by**: Agnès Caille

SOMMAIRE

I. Background 3

II. Objectives 3

1. Primary objective 3

2. Secondary objectives 3

III. Methodology 3

1. Design 4

2. Population 4

3. Outcomes 4

a) Primary outcome 4

b) Secondary outcomes 5

4. Procedures 5

5. Sample size 6

IV. Statistical considerations 6

1. General principles 6

a) Missing data 6

b) Significance level 6

c) Analysis population definition 6

2. Baseline characteristics 6

3. Statistical analysis of the primary outcome 6

4. Statistical analysis of the secondary outcomes 6

5. Additional analyses 7

6. Software 7

1. Background

Severe acute respiratory syndrome coronavirus 2 is an emergent coronavirus, first reported in Wuhan, China 2019. So far, the virus has infected more than 1.3 million people all around the world and caused death of more than 74 000 persons (13th April 2020). Up to 20% of hospitalized patients need an admission in intensive care medicine to receive ventilation support and the vast majority experienced acute respiratory distress syndrome (ARDS).

To date, there is no efficient therapeutics to prevent or treat COVID-19 related respiratory failure. Identifying a drug is a major concern and a public health emergency. The pathogenesis of COVID-19 encompasses a “cytokine storm” which includes pro-inflammatory interleukins (IL-1β, IL-6) and tumor necrosis factor (TNF-α). Several studies suggest that patients who develop severe respiratory forms of COVID-19 have a deleterious pulmonary and systemic inflammatory cascade. Predictors of mortality from a recent retrospective, multicentre study of 150 confirmed COVID-19 cases in Wuhan, China, included elevated ferritin and elevated C-Reactive Protein (mean 126 mg/L in non survivors vs. 34 mg/L in survivors) suggesting that mortality might be due to virally driven hyperacute inflammation. It is well known that circulating CRP is produced by hepatocytes under regulatory control from circulating cytokines, in particular IL-6 and IL1β.

Therefore, we suggest that IL-1β is a potentially therapeutic target to break inflammatory process and to treat patient at risk for developing severe ARDS related to COVID-19. As a recombinant anti-human IL-1 receptor treatment, Anakinra can specifically binds IL-1R and inhibit signal transduction. For a decade, Anakinra is mainly used to treat patients with rheumatoid arthritis or auto-inflammatory diseases and its safety profile is favorable in such indications. Moreover, subgroup analysis of data from a phase III randomized controlled trial of IL-1 blockade (Anakinra) in sepsis, showed a significant survival benefit in patients with hyperinflammation, without increased adverse events as compared to placebo. Several multicentre, randomized controlled trials of tocilizumab (IL- 6 receptor blockade) or sarilumab (IL-6 neutralisation) are ongoing in many countries for COVID-19 patients.

We, therefore, aimed to study the efficacy and safety of Anakinra plus optimized standard of care compared to optimized standard of care alone, in patients with COVID-19 with aggravating respiratory symptoms and inflammatory component in a multicenter randomized clinical trial.

1. Objectives
2. Primary objective

The main objective of the ANACONDA-COVID-19 trial is to assess the efficacy of Anakinra + optimized Standard of Care (oSOC) as compared to oSOC alone on the condition of patients with COVID-19 infection and worsening respiratory symptoms.

1. Secondary objectives

To assess the efficacy of Anakinra + oSOC as compared to oSOC alone on:

Treatment success (same definition than primary outcome) up to Day 28

Patient’s condition as defined by the OMS 7 point scale up to Day 28

Patient’s mortality up to Day 28

Patient’s admission in ICU

Pulmonary function (need for ventilator support, SP0_2_, PaO_2_/FiO_2_) up to Day 28

Inflammation parameters up to Day 28

To evaluate the safety profile of Anakinra

1. Methodology
2. Design

ANACONDA-COVID-19 study is a French multicentre, open-label, randomized, and controlled superiority trial comparing the administration of optimized standard of care and Anakinra versus optimized standard of care alone in patients hospitalized in a medical unit with COVID-19.

1. Population

Inclusion criteria:

- Male or female≥ 18 years of age
- Written informed consent of the patient or a proxy
- Ability for participant to comply with the requirements of the study
- Hospitalized patient with COVID-19 defined as
  - Positive SARS-CoV2 RT-PCR or
  - Typical COVID-19 Radiographic infiltrates on the CT scan other non COVID-19 diagnosis ruled out.
- Patient with respiratory symptoms and requirement of oxygen therapy as defined:
  - Oxygen therapy ≥ 4L/min to maintain Sp0_2_>92% and respiratory rate ≥ 24/min or
  - Patients under oxygen ≥ 1L/min and presenting worsening of oxygen requirement defined by an increase of oxygen therapy ≥ 2L/min to maintain Sp0_2_ > 92%.
- Inflammatory component C-Reactive Protein ≥ 50mg/L.
- Patients within the first 20 days from the onset of the first COVID-19 symptoms
- Probabilistic antibiotics therapy according to local practice

Non-inclusion criteria:

- Respiratory failure related to other cause than COVID-19
- Patients requiring mechanical ventilation at inclusion or requiring oxygen therapy equal or more than 11 liters per min to maintain Sp0_2_ > 92%
- Infectious diseases such as severe bacterial infections, aspergillosis, HIV, active HCV, active HBV, active tuberculosis
- Contra indication to anti-IL1 receptor
  - Known hypersensitivity to Anakinra
  - Absolute neutrophil count (ANC)< 1500/mm3
  - Liver cirrhosis score de Child-Pugh class C
  - Live or attenuated vaccine in the past 8 weeks
  - Pregnant or breast-feeding women
- Patients with either legally protected status or who have been deprived of their freedom
- Patient included in other interventional therapeutic research (e.g. = concurrent participation in French CoVID-19 is accepted)
- Patients who have received previous treatment by anti-IL6R, anti-IL-6, anti-IL1R, anti-IL1 or anti-TNF_α_ within 21 days preceding inclusion.
- Absence of Health Insurance
- Existence of any life-threatening co-morbidity or any other medical condition which, in the opinion of the investigator, makes the patient unsuitable for inclusion.

1. Outcomes
2. Primary outcome

The primary endpoint is treatment success at Day 14, defined as a patient alive and not requiring any of the following: Invasive mechanical ventilation (IMV) or Extracorporeal membrane oxygenation (ECMO).

1. Secondary outcomes

Efficacy outcomes:

Treatment success (same definition as the Primary outcome) at Day 3, Day 10 and Day 28.

OMS progression scale (on a 7-point ordinal scale): at Day 3, Day 10, Day 14 and Day 28:

1. Not hospitalized, no limitations on activities

2. Not hospitalized, limitation on activities;

3. Hospitalized, not requiring supplemental oxygen;

4. Hospitalized, requiring supplemental oxygen;

5. Hospitalized, on non-invasive ventilation or high flow oxygen devices;

6. Hospitalized, on invasive mechanical ventilation or ECMO;

7. Death.

Overall survival at Day 3, Day 10, Day 14 and Day 28

Time to ICU admission

Time to ventilatory support (ECMO, invasive mechanical ventilation, non-invasive ventilation, high flow oxygen therapy)

Change in NEWs score from baseline to Day 3, Day 10, Day 14 and Day 28

Change in inflammatory parameters (CRP, ferritin, D-dimer, fibrinogen, lymphocytes count, platelet count) from baseline to Day 3, Day 10, Day 14 and Day 28

Hospital length of stay: Time from inclusion to hospital discharge

For those admitted in ICU:

Need for Vasopressors (yes or no)

If IMV, Evolution of SpO2/FIO2

If IMV, Evolution of PaO2/FiO2 ratio

ICU length of stay: Time from admission in ICU to ICU discharge

The safety outcomes: Occurrence of serious adverse events during the study, including infection (bacterial, parasitic, mycotic and viral infection), septic shock, Ankanira hypersensitivity, hepatic damages (SGOT/SGPT, alkaline phosphatase, gammaGT) and neutropenia (Blood count).

1. Procedures

After consent, eligible patients will be randomly allocated with a 1:1 ratio to either:

- Anakinra plus optimized standard of care: The patients will receive Intraveinous injection (IV) of Anakinra 400mg/day (100mg IV every 6 hours) at Day 1, 2 and 3. From Day 4 to Day 10, the patient will receive IV injection of Anakinra 200mg/day (100mg every 12 hours). The total duration of Anakinra is 10 Days.
- or optimized standard of care alone.

Randomization (Day 0) will be stratified on baseline CRP concentration (< 150 *vs.* ≥ 150 mg/L), baseline requirement of oxygen therapy to maintain Sp0_2_ over 92% (3-6 liters per min *vs.* 7-10 liters per min), and corticosteroid therapy at baseline (< *vs.* ≥ 0.5mg/kg/day prednisone) (yes *vs.* no). Patients will be followed up to day 28. Baseline measurements will include clinical, radiological and biological measurements. Patients will be followed daily during hospitalization and study visits will be performed at Day 3, Day 10, Day 14 and Day 28.

1. Sample size

We plan to recruit 120 patients per group thus a total of 240 patients. With alpha set to 5%, beta to 20%, a bilateral test, an estimated proportion of success in the control group of 80%, 216 patients are needed to show an absolute increase in the proportion of success of 13 percentage points in the experimental group (i.e. 93% of success). With one interim analysis, using Pocock’s approach, and applying a 1.11 inflation factor on the sample size, 240 patients are needed to achieve the 80% pre specified power.

1. Statistical considerations
2. General principles
3. Missing data

Missing data for the primary outcome will be considered as a treatment failure, whatever the study group. Nevertheless, in this study, the likelihood that patients will be lost to follow-up is small. No imputation of missing data will be performed on the secondary outcomes.

1. Significance level

The significance level associated with the interim and final analysis will be 0.05 because no interim analysis was performed.

1. Analysis population definition

Each patient will remain in the group assigned by randomisation, regardless of subsequent events.

1. Baseline characteristics

Baseline characteristics will be reported per group using descriptive statistics. No statistical test will be performed on baseline measures.

1. Statistical analysis of the primary outcome

The number and proportion of patients with treatment success at Day 14 will be reported in each group. We will estimate the between group risk difference, both point estimate and 95% confidence interval will be reported. The comparison of the proportion of treatment success between the two groups will be performed using a chi-square test. We will also estimate the adjusted between group risk difference using a linear model (identity link function), adjustment variables will be stratification variables for randomization i.e. baseline CRP value (< 150 *vs.* ≥ 150 mg/l), baseline requirement of oxygen therapy to maintain Sp0_2_ over 92% (3-6 liters per min *vs.* 7-10 liters per min) and corticosteroid therapy at baseline (≥ 0.5mg/kg: yes *vs* no).

1. Statistical analysis of the secondary outcomes

- Treatment success (same definition as the Primary outcome) at Day 3, Day 7 and Day 28 will be analysed in the same manner as the primary outcome.
- OMS progression scale (on a 7-point ordinal scale): at Day 3, 10, 14 and 28. The number and proportion of patients in each category will be reported. Between-group comparison will be performed using non parametric Cochran-Armitage tests.
- Overall survival will be summarized using Kaplan-Meier curves and compared with the use of log-rank test.
- Time to ICU admission and time to ventilatory support (ECMO, invasive mechanical ventilation, non-invasive ventilation, high flow oxygen therapy) will be analyzed using the competing risk approach, with death as competing risk.
- Change from baseline to Day3, Day10, Day 14 and Day28 in NEWs score and inflammatory parameters will be described graphically using boxplots (one by study group at each visit) depicted on the same graph; changes over time will be compared between the two groups using mixed linear models, after data transformation if necessary
- Hospital length of stay: Time from inclusion to hospital discharge will be compared between the two groups using the competing risk approach, with death as competing risk
- Number and proportion of patients with at least one serious adverse event during the study, particularly bacterial infection, septic shock, hepatic damage and neutropenia will be provided by study group and compared using a chi-square test
- For those admitted in ICU only descriptive statistics will be provided:
- Number and proportion of patients with need for vasopressors will be reported.
- If IMV, Evolution of SpO_2_/FIO_2_ and PaO_2_/FiO_2_ ratio will be described graphically using boxplots (one by study group at each visit) depicted on the same graph.
- ICU length of stay: Time from admission in ICU to ICU discharge will be described.

1. Additional analyses

Subgroup analyses

We plan to perform subgroup analyses on the stratification variables for randomization:

- Baseline CRP value <150 *vs.* ≥ 150 mg/l: yes *vs* no.
- Baseline requirement of oxygen therapy to maintain Sp0_2_ over 92% (3-6 liters per min *vs.* 7-10 liters per min: yes *vs* no).
- Corticosteroid therapy at baseline (< *vs.* ≥ 0.5mg/kg prednisone: yes *vs* no).

Other subgroup analysis will include:

- Ddimers count <2 000 *vs.* ≥ 2000 ng/ml: yes *vs* no.
- Lymphocytes count <500/mm^3^ *vs.* ≥ 500/mm^3^: yes *vs* no.

We will use of a linear model with an identity link function to estimate the risk differences in each subgroup as well as interaction p-values.

1. Software

SAS version 9.4, R version 3.3.1.
