# Supplementary material for: Efficacy and safety of anakinra in adults presenting deteriorating respiratory symptoms from COVID-19: A randomized controlled trial
Source: PLoS One. 2022 Aug 4;17(8):e0269065. doi: 10.1371/journal.pone.0269065 (PMC9351999; doi:10.1371/journal.pone.0269065)
Supplement: S1 Table — (DOCX) [file pone.0269065.s004.docx]

**Table S1: Anakinra administration, number of doses administered per patient and reasons for incomplete regimen**

| **Nb of complete doses** | **n (%)** | **Reasons** |
| --- | --- | --- |
| 0 | 1 (2.7) | Consent withdrawal |
| 1 | 2 (5.4) | AE + death ; death |
| 2 | 3 (8.1) | Death ; decision of sponsor ; dose at 300mg (clearance of creatinine <30) |
| 4 | 2 (5.4) | AE ; decision of sponsor |
| 5 | 2 (5.4) | Death ; Transfer |
| 6 | 2 (5.4) | Dose adaptation (DFG <30) ; decision of clinician |
| 7 | 1 (2.7) | AE |
| 8 | 1 (2.7) | Hospital discharge |
| 9 | 8 (21.6) | 4 Logistic problems, 3 not known, 1 hospital discharge |
| 10 | 15 (40.5) | - |
