# Supplementary material for: Efficacy and safety of anakinra in adults presenting deteriorating respiratory symptoms from COVID-19: A randomized controlled trial
Source: PLoS One. 2022 Aug 4;17(8):e0269065. doi: 10.1371/journal.pone.0269065 (PMC9351999; doi:10.1371/journal.pone.0269065)
Supplement: S2 Table — (DOCX) [file pone.0269065.s005.docx]

**Table S2: Corticoids administered at inclusion or during follow-up**

|  | **Anakinra plus optimized standard of care group**  **(n_1_=37)** | **Optimized standard of care group**  **(n_2_=34)** |
| --- | --- | --- |
| Corticosteroids at inclusion or after | 28 (75.7) | 30 (88.2) |
| Mean daily dose of corticosteroids administered at inclusion or after (equivalent prednisone) , *n_1_=28, n_2_=30* | 64.6 ± 48.5 | 60.0 ± 30.5 |
|  | 50.0 [40.0 ; 80.1] | 46.0 [40.0 ; 75.0] |

*n (%) for qualitative variables ; Mean ± standard deviation; median [1^st^quartile; 3^rd^ quartile] for continuous variables*
