# Supplementary material for: Efficacy and safety of anakinra in adults presenting deteriorating respiratory symptoms from COVID-19: A randomized controlled trial
Source: PLoS One. 2022 Aug 4;17(8):e0269065. doi: 10.1371/journal.pone.0269065 (PMC9351999; doi:10.1371/journal.pone.0269065)
Supplement: S1 Fig — (DOCX) [file pone.0269065.s006.docx]

**Figure S2: Subgroup analyses of the primary outcome**


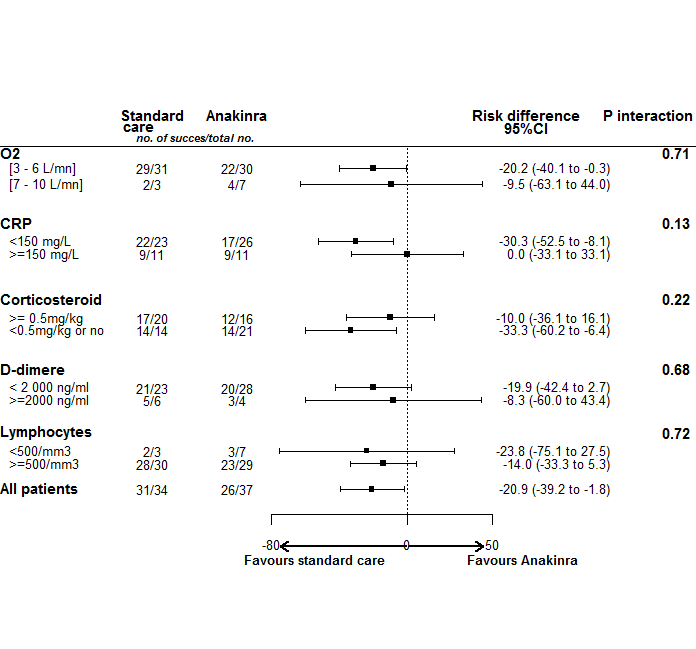


Risk difference was defined as the between-group difference (Anakinra group minus Standard care group) in percentage points.
