# Supplementary material for: Efficacy and safety of anakinra in adults presenting deteriorating respiratory symptoms from COVID-19: A randomized controlled trial
Source: PLoS One. 2022 Aug 4;17(8):e0269065. doi: 10.1371/journal.pone.0269065 (PMC9351999; doi:10.1371/journal.pone.0269065)
Supplement: S2 Fig — (DOCX) [file pone.0269065.s007.docx]

**Figure S3: Evolution of NEWs score across the 28-day follow-up**


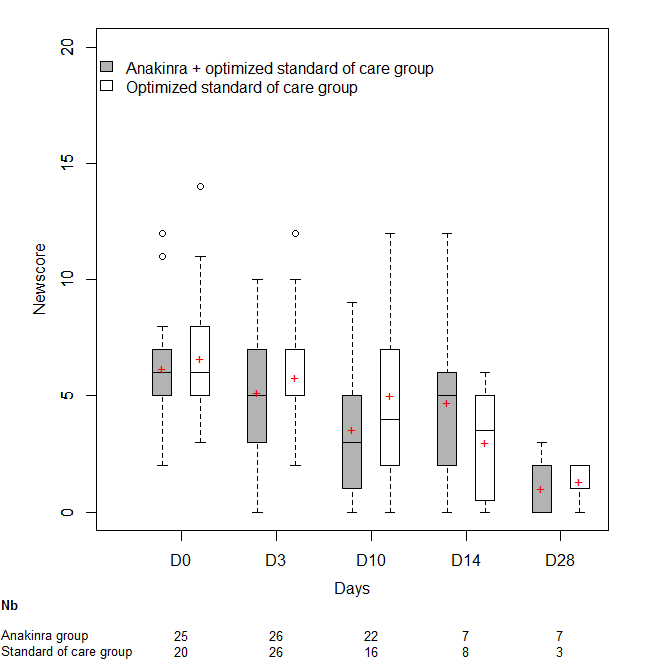


| **Effect** | **Parameter & 95%CI** | **p** |
| --- | --- | --- |
| Intercept | 6.47 [5.66 ; 7.28] | <.0001 |
| Time effect, *days* | -0.20 [-0.29 ; -0.12] | <.0001 |
| Treatment effect, *Anakinra vs standard care* | -0.65 [-1.74 ; 0.44] | 0.24 |
| **Time by treatment interaction** | **0.02 [-0.08 ; 0.13]** | **0.67** |

** Death was not taken into account in this analysis.*

Between group difference in NEW score evolution was assessed through time by treatment interaction term.
