# Supplementary material for: Efficacy and safety of anakinra in adults presenting deteriorating respiratory symptoms from COVID-19: A randomized controlled trial
Source: PLoS One. 2022 Aug 4;17(8):e0269065. doi: 10.1371/journal.pone.0269065 (PMC9351999; doi:10.1371/journal.pone.0269065)
Supplement: S3 Fig — (DOCX) [file pone.0269065.s008.docx]

**Figure S4: Evolution of inflammatory parameters across the 28-day follow-up**


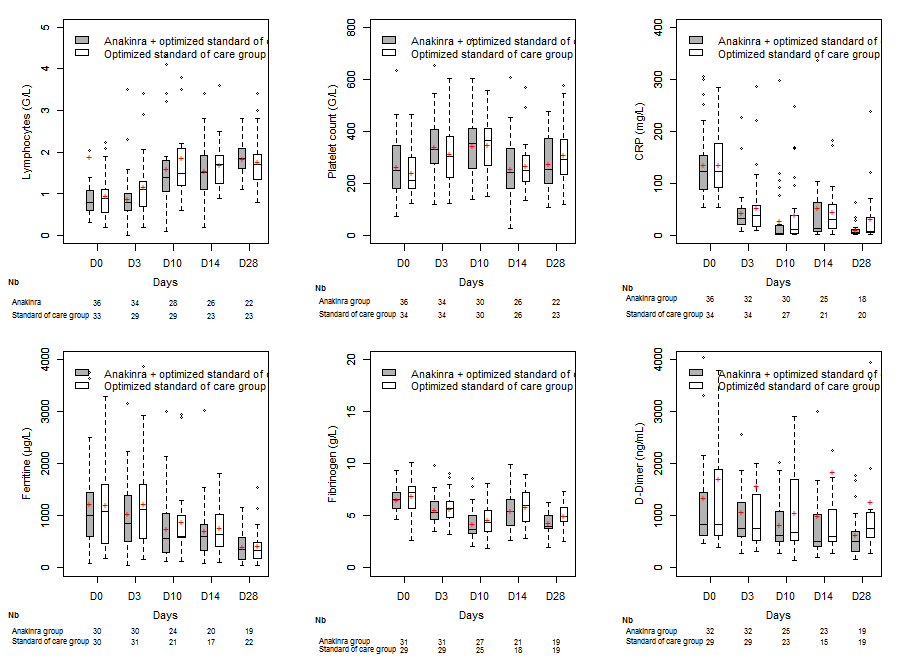


| **CRP** | **Parameter & 95%CI** | **p** |
| --- | --- | --- |
| Intercept | 4.13 [3.83 ; 4.42] | <.0001 |
| Time effect, *days* | -0.07 [-0.09 ; -0.04] | <.0001 |
| Treatment effect, *Anakinra vs standard care* | -0.10 [-0.51 ; 0.31] | 0.62 |
| **Time by treatment interaction** | -0.02 [-0.06 ; 0.01] | 0.22 |

| **Ferritin** | **Parameter & 95%CI** | **p** |
| --- | --- | --- |
| Intercept | 6.91 [6.65 ; 7.17] | <.0001 |
| Time effect, *days* | -0.03 [-0.04 ; -0.03] | <.0001 |
| Treatment effect, *Anakinra vs standard care* | -0.14 [-0.51 ; 0.22] | 0.43 |
| **Time by treatment interaction** | -0.005 [-0.02 ; 0.007] | 0.40 |

| **D-Dimer** | **Parameter & 95%CI** | **p** |
| --- | --- | --- |
| Intercept | 6.96 [6.73 ; 7.19] | <.0001 |
| Time effect, *days* | -0.01 [-0.02 ; 0.001] | 0.07 |
| Treatment effect, *Anakinra vs standard care* | -0.10 [-0.42 ; 0.21] | 0.52 |
| **Time by treatment interaction** | -0.01 [-0.02 ; 0.006] | 0.24 |

| **Fibrinogen** | **Parameter & 95%CI** | **p** |
| --- | --- | --- |
| Intercept | 6.13 [5.68 ; 6.58] | <.0001 |
| Time effect, *days* | -0.05 [-0.08 ; -0.02] | <.0001 |
| Treatment effect, *Anakinra vs standard care* | -0.15 [-0.78 ; 0.47] | 0.64 |
| **Time by treatment interaction** | -0.007 [-0.05 ; 0.03] | 0.69 |

| **Lymphocytes count** | **Parameter & 95%CI** | **p** |
| --- | --- | --- |
| Intercept | -0.03 [-0.26 ; 0.20] | 0.80 |
| Time effect, *days* | 0.02 [0.01 ; 0.03] | <.0001 |
| Treatment effect, *Anakinra vs standard care* | -0.15 [-0.47 ; 0.17] | 0.34 |
| **Time by treatment interaction** | 0.005 [-0.004 ; 0.01] | 0.29 |

| **Platelet count** | **Parameter & 95%CI** | **p** |
| --- | --- | --- |
| Intercept | 5.58 [5.45 ; 5.71] | <.0001 |
| Time effect, *days* | 0.005 [-0.002 ; 0.01] | 0.16 |
| Treatment effect, *Anakinra vs standard care* | 0.04 [-0.13 ; 0.22] | 0.63 |
| **Time by treatment interaction** | -0.008 [-0.02 ; 0.002] | 0.10 |

Between-group differences in inflammatory parameters evolutions were assessed through time by treatment interaction term.
